# Proteomic Analysis Infers Optimized ATP‐Production in Guard Cell Mitochondria

**DOI:** 10.1111/ppl.70529

**Published:** 2025-09-26

**Authors:** Noah Ditz, Markus Niehaus, Nieves Medina Escobar, Marco Herde, Holger Eubel

**Affiliations:** ^1^ Institute of Plant Genetics Leibniz University Hannover Hannover Germany; ^2^ Institute of Plant Nutrition Leibniz University Hannover Hannover Germany

**Keywords:** amino acid catabolism, energy metabolism, guard cells, mitochondria, proteomics

## Abstract

Guard cells are highly specialised leaf cells which regulate leaf transpiration and carbon fixation. As such, they are instrumental in balancing water use efficiency and photosynthetic activity. This unique function of guard cells requires them to quickly accumulate solutes for ATP‐dependent stomatal opening, but how this affects mitochondrial energy metabolism remains elusive. Using cell‐type‐specific affinity purification of *Arabidopsis thaliana* guard cell and mesophyll cell mitochondria, we aim at unravelling the enzymatic configuration of guard cell mitochondria in order to provide a first glance at the metabolic properties of these specialised organelles. When compared to their mesophyll cell counterparts, the use of alternative substrates for NADH production, a shift away from non‐proton pumping respiratory enzymes, and a lower NADH re‐oxidation rate suggest a specialised mode of ATP production in guard cell mitochondria. In addition, a lowered abundance of the mitochondrial import machinery also indirectly implies lower protein turnover rates in these organelles.

## 1 Introduction

Mitochondria are essential organelles of carbon metabolism and energy conversion. The tricarboxylic acid (TCA) cycle and oxidative phosphorylation are mitochondria‐localized steps of cellular respiration and provide the bulk of ATP for driving most cellular processes. Mature mesophyll chloroplasts are an exception to this since they can support their own ATP demands and do not import appreciable amounts of mitochondria‐derived ATP. Still, mitochondria support basic chloroplast functions during the day. They serve as sink organelles for surplus reduction equivalents in the leaf mesophyll (Podgórska et al. 2020) and produce precursor molecules such as citrate and 2‐oxoglutarate (2‐OG) for nitrogen assimilation. Hosting the NAD‐dependent oxidation of glycine by the glycine decarboxylase complex (GDC) and the ensuing biosynthesis of serine, they also participate in the photorespiratory (PR) pathway. Since GDC activity produces large amounts of NADH during the day, it is hypothesized that the TCA cycle changes flux over the diurnal cycle in autotrophic plant tissues (Sweetlove et al. 2010). In addition to the different TCA cycle modes, electron transfer in the plant respiratory chain also shows some degree of flexibility. Alternative respiratory enzymes, such as the type II NAD(P)H dehydrogenases NDA, NDB, and NDC, as well as alternative oxidase (AOX) isoforms prevent over‐reduction of respiratory chain components in times of ADP shortage (Rasmusson et al. 2004; Gardeström and Igamberdiev 2016). Plant uncoupling mitochondrial proteins (PUMPs) also operate in this context, since they are able to dissipate the proton gradient across the inner mitochondrial membrane independently from the activity of the ATP synthase complex (Smith et al. 2004). The special features of mesophyll cell mitochondria required for efficient operation in the context of photosynthesis thus shape a unique metabolism that is clearly distinguishable from its counterpart in heterotrophic tissues (Lee et al. 2008).

In contrast to mesophyll cells, the scope and aim of the photosynthetic activity within guard cells are still a matter of debate. Guard cell chloroplast functions are unique and differ from their mesophyll cell counterparts in respect to starch synthesis and degradation patterns. Since stomatal movement heavily depends on guard cell starch content, starch is produced when stomata close and degraded when they open (Robinson and Preiss 1985; Santelia and Lunn 2017). Accumulation of starch in guard cells is thus observed during the night, markedly differing from the situation in mesophyll cells, where it is produced during the day and mostly used up during the course of the night. Starch biosynthesis in guard cells is partly fueled by carbon originating from the mesophyll and mitochondria‐derived ATP (Lim et al. 2022; Flütsch et al. 2022). Taking into account these differences between guard cell chloroplasts and mesophyll cell chloroplasts, as well as the integration of mitochondrial and chloroplast metabolism, the roles of guard cell mitochondria in supporting photosynthesis are expected to differ from those of their mesophyll cell counterparts.

Mitochondrial activity in guard cells also benefits other cellular functions. Stomatal movement requires active transport of ions and solutes across the plasma membrane and tonoplast. For stomatal opening, the water potential of guard cells is reduced by the action of proton‐pumping H^+^‐ATPases, leading to a hyperpolarization of the plasma membrane and an influx of K^+^ ions into the cytosol. This is accompanied by negatively charged counter‐ions, predominantly Cl^−^ and malate^2−^. The increased osmolality of guard cells entails water influx, thus increasing turgor, bending of the cells, and opening of the stomatal pore. The massive amounts of ATP required for this process seem to stem from different sources, including photophosphorylation, glycolysis, and oxidative phosphorylation. It is, however, unclear how much each of these processes contributes toward stomatal opening and how the balance of energetic contributions between chloroplasts, cytosol, and mitochondria changes in response to the conditions (Schwartz and Zeiger 1984; Zhao and Assmann 2011; Tominaga et al. 2001; Mawson 1993). Another process controlling the water potential in the guard cell cytoplasm is the interconversion of organic metabolites. In response to blue light, starch is degraded early in the day to produce sucrose and other organic compounds (Lasceve et al. 1997), thus adding to an already decreased guard cell water potential resulting from the ion/solute transport into the cells. For stomatal closing, these metabolites are either degraded and subsequently channeled into the TCA cycle or are used for starch synthesis in chloroplasts.

Core mitochondrial processes, such as the TCA‐cycle and oxidative phosphorylation, are therefore essential for stomatal movement. Considering the special features of guard cell physiology, such as the high demand for ATP and the involvement of TCA‐cycle intermediates in stomatal movement, it seems likely that guard cell mitochondrial functions reflect their unique environment and the physiological challenges this brings along. This notion is supported by the relatively high number of these organelles in guard cells (Allaway and Setterfield 1972; Willmer and Fricker 1996). Analyzing guard cell mitochondrial metabolism is therefore also expected to benefit our understanding of guard cell metabolism *in toto*.

Until recently, biochemical appraisal of plant mitochondria and their functions relied exclusively on bulk‐phase fractions. Mitochondria isolated from plant organs (such as leaves, roots, stems, flowers) are best described as pool fractions, since the starting material contains more than a single cell type. Properties of low‐abundant, specialized mitochondria, including their proteomes and functional parameters, are often completely absorbed in pool fractions. It is therefore impossible to quantify how mitochondrial functions differ among the cell types contributing to any given plant organ. Using a mitochondria affinity purification (Mito‐AP) approach (Niehaus et al. 2020), allowing the selective isolation of guard cell and mesophyll cell mitochondria, we here provide first insights into the functional specialization of guard cell mitochondria and how their properties set them apart from mesophyll cell mitochondria. The differences between the two mitochondrial populations observed here provide new insights into the physiology of mitochondria from two fundamentally different cellular environments within the same plant organ.

## 2 Materials and Methods

### 2.1 Cloning

A MoClo (Engler et al. 2014) based protocol similar to the strategy presented in Niehaus et al. (2020) was used for cloning of a construct allowing the expression of the Twin‐Strep‐tag:GFP:ADAPTER protein under the control of a guard cell specific promoter (H972). In detail, the DNA encoding the Twin‐Strep‐tag:GFP:ADAPTER was amplified with P2235 and P2236 using the original H500 template (Niehaus et al. 2020). The resulting PCR product was combined in a cut/ligation reaction (using BsaI and T4 ligase) with pTEI070 (Gantner et al. 2018), containing a 1 kb fragment upstream of AT1G22690 (pGC1) (Yang et al. 2008) as a strong guard cell specific promoter and pICH41421 (3′UTR, polyadenylation signal/terminator, nos) (Engler et al. 2014) as well as the recipient vector pICH47732 (Engler et al. 2014). The resulting construct (H969) was combined with V166 (Niehaus et al. 2020) and the level 2 recipient vector pAGM4723 (Engler et al. 2014) in a cut/ligation reaction (using BpiI and T4 ligase) to generate the final construct H972. For mesophyll‐specific expression, the constructs H970 (level 1 vector) as well as H973 (level 2 vector) were cloned with the same strategy using the vector pTEI071 (Gantner et al. 2018) containing a 1 kb fragment upstream of Arabidopsis AT1G29910 (CAB3) (Susek et al. 1993), instead of pTEI070.

### 2.2 Plant Material and Cultivation

*Arabidopsis thaliana* Col‐0 wild type (WT) plants, as well as homozygous T_2_ H500, H973, and H972 plants, were grown in soil (Einheitserde Special Profisubstrat, Patzer Erden GmbH) under a photosynthetically active photon flux density (PPFD) of 120 μmol m^−2^ s^−1^ (using Philips F25T8/TL841 fluorescence lamps) under long day conditions (16/8 h light/dark) for 4 weeks. Temperature was set to 22°C in the light and 20°C in the dark, while relative humidity was kept at 65%.

### 2.3 Confocal Microscopy

Leaf discs of four‐week‐old H972 and H973 plants were analysed with a Leica SP8 confocal fluorescence microscope equipped with a HC PL APO CS2 40 × 1.10 water immersion objective (Leica Microsystems), using an excitation wavelength of 488 nm (green fluorescent protein, GFP) and 552 nm (chloroplast autofluorescence). The emission wavelength was collected between 502 and 516 nm (GFP) and between 658 and 708 nm (chloroplast autofluorescence). Images were processed using the Leica Application Suite X (LAS X) software version 3.10.0. Z‐stack projections displaying epidermal or mesophyll cells were deduced from the same Z‐stack covering both tissues.

### 2.4 Preparation of Magnetic Beads for Isolation of Mitochondria

NHS‐activated magnetic beads (Thermo Fisher Scientific) were coated with Strep‐Tactin (IBA Life Science GmbH) according to the manufacturer's instructions with minor modifications. In detail, 4 mg of beads were washed once with ice‐cold 1 mM HCl by vortexing for 15 s. Beads were pelleted for 2 min in a magnetic field, and the supernatant was discarded before beads were supplemented with Strep‐Tactin solution (2 mg ml^−1^ in 0.2 M sodium hydrogen carbonate, pH 8.5). Beads were vortexed for 30 s and incubated in an overhead shaker for 2 h at room temperature. Beads were vortexed for 15 s every 5 min during the first 30 min of incubation. Afterwards, beads were vortexed for 15 s every 15 min. Coated beads were washed twice in 0.1 M glycine (pH 2.0) and once in ultrapure water by vortexing for 15 s. Between each step, beads were pelleted in a magnetic field for 2 min. Uncoupled NHS groups were quenched with 3 M ethanolamine (pH 9.0) for 2 h at room temperature in an overhead shaker. Beads were then washed once in ultrapure water, followed by three wash steps in 0.2 M sodium hydrogen carbonate (pH 8.5). Washed beads were stored as 400 μL ‘bead stocks’ in 0.05% [w/v] sodium azide and 0.2 M sodium hydrogen carbonate (pH 8.5) at 4°C for a maximum of 1 week.

### 2.5 Affinity Purification of Mitochondria

Isolation of mitochondria was performed at 4°C or on ice using pre‐cooled materials and buffers. For shotgun mass spectrometry (MS) sample preparation, 50 μL of bead stock was used, whereas 250 μL of beads was required for BN‐PAGE and NADH‐oxidation assays. Beads were equilibrated three times in 1 mL wash buffer (100 mM sucrose, 200 mM NaCl, 1.5 mM MgCl_2_, 1 mM DTT, 50 mM HEPES‐NaOH, pH 8.0) and finally resuspended in 500 μL of the same solution. One gram of rosette leaf material was harvested 2 h after the onset of illumination and homogenized for 2 min in a mortar with 2 mL extraction buffer (wash buffer with 3.2% [v/v] freshly added BioLock; IBA Lifescience GmbH) and a spatula of sea sand. The crude extract was centrifuged at 1000 *g* for 5 min at 4°C. Subsequently, 1.5 mL of the supernatant was mixed with 0.5 mL of equilibrated beads. The mixture was incubated for 5 min in an overhead shaker before beads were pelleted in a magnetic field for 2 min. The supernatant was discarded and beads were resuspended in 1 mL wash buffer by gentle manual inverting for 30 s, followed by incubation on an overhead shaker for 3 min. Beads were subsequently pelleted on a magnet for 2 min, after which the supernatant was discarded and beads were resuspended in 1 mL wash buffer. After 3 min incubation in an overhead shaker, this procedure was repeated two further times for a total of three wash cycles.

### 2.6 Shotgun Proteomics

For a detailed description of the proteomic workflow, please consult the supplemental section. In brief, AP‐purified mitochondria bound to magnetic beads were submitted to the SP3 sample preparation and trypsin digestion workflow (Hughes et al. 2019; Mikulášek et al. 2021). Two hundred nanograms of desalted peptides were subsequently separated in a nanoElute2 UHPLC (Bruker) using a 60 min 2% [v/v] to 37% [v/v] acetonitrile (ACN) in 0.1% [v/v] formic acid (FA) gradient over a C18 analytical column (Aurora Ultimate 25 cm × 75 μm, 1.7 μm particle size, 120 Å pore size; IonOpticks). Peptide analysis was performed with a timsTOF Pro (Bruker) using a short proteomics method with 0.5 s cycle time. Acquired MS/MS spectra were queried against an in‐house modified TAIR10 database containing protein sequences considering organellar RNA editing using the MaxQuant software version 2.6.3.0 (Cox and Mann 2008), followed by quantitative statistical analysis using the Perseus software version 2.1.2.0 (Tyanova et al. 2016).

### 2.7 Blue‐Native Polyacrylamide Gel‐Electrophoresis (BN‐PAGE) Followed by LC–MS/MS Analysis

Solubilization of mitochondrial protein was achieved by resuspending bead‐bound mitochondria in 100 μL digitonin solubilization buffer (150 mM potassium acetate, 10% [v/v] glycerol, 5% [g/v] digitonin, 30 mM HEPES‐HCl, pH 7.4). After 20 min incubation on ice, beads were pelleted for 2 min in a magnetic field, and the supernatant was centrifuged at 18300 *g* for 10 min at 4°C. Protein concentrations of the supernatants were determined according to Bradford and adjusted to 1 μg μL^−1^ using solubilization buffer without digitonin. Samples were supplemented with 5% [v/v] Serva Blue‐G (750 mM aminocaproic acid (ACA), 5% [w/v] Coomassie 250 G), and 185 μg of protein was loaded on a linear polyacrylamide gradient gel (4.5% T, 6.25% C to 16% T, 6.25% C in 300 mM ACA and 30 mM Bis‐Tris–HCl, pH 7) overlaid with a 4% T, 6.25% C sample gel. Separation of protein assemblies was carried out at 4°C using a voltage of 100 V for the first 45 min, followed by 11 h at 500 V. The current was limited to 15 mA. Gels were stained according to Neuhoff et al. (1988), and lanes were cut into 39 fractions of equal size (10 mm broad, 4 mm high, 1.5 mm thick).

Each fraction was subjected to tryptic in‐gel digestion as described in Klodmann et al. (2010). Tryptic peptides were vacuum dried and resuspended in 20 μL 0.5% [v/v] acetic acid for subsequent desalting using self‐made C18 filter tips. Preparation of filter tips and desalting was carried out according to Rappsilber et al. (2007), with minor modifications. Six discs of Empore Solid Phase Extraction (SPE) C18 material (0.5 mm thickness, 12 μm pore size; purchased by Merck KGaA) were pressed into 200 μL pipette tips. Tips were washed once with 50 μL methanol, followed by a wash with 50 μL 80% [v/v] ACN in 0.5% [v/v] acetic acid. Equilibration was achieved with 50 μL of 0.5% [v/v] acetic acid before samples were loaded on filter tips. Bound peptides were washed once with 50 μL of 0.5% [v/v] acetic acid and subsequently eluted in three steps, each time using 40 μL of increasing ACN concentrations (30% [v/v] ACN, 50% [v/v] ACN, 80% [v/v] ACN, each in 0.5% [v/v] acetic acid). Eluates were pooled in the same well of a 96‐microtiter plate and subsequently vacuum‐dried.

Desalted peptides were resuspended in 12 μL of 0.1% [v/v] FA and incubated in an ultrasonication bath for 10 min before being stored in glass vials at 4°C in an autosampler of a nanoElute2 HPLC coupled to a timsTOF Pro MS (both Bruker). Three microliters of peptide suspension were injected into a 20 μL sample loop before being loaded onto a 15 cm reverse phase Aurora Elite CSI analytical column (IonOpticks) with an inner diameter of 75 μm, a particle size of 1.7 μm, and a pore size of 120 Å. Separation took place at a flow rate of 500 nL min^−1^ and a column temperature of 50°C using a 20 min ACN gradient from 2% [v/v] to 37% [v/v] ACN (in the presence of 0.1% [v/v] FA). Afterwards, the ACN concentration rose to 95% [v/v] over 30 s and was kept at this value for 2 min to clear the column of highly hydrophobic compounds. Ionization of peptides was achieved at a temperature of 180°C and a capillary voltage of 1600 V. The scan range was defined from 100 to 1700 m/z using the PASEF scan mode with ion mobilities (expressed as 1/K0) ranging from 0.85 to 1.3 V s^−1^ cm^−2^. The ramp time was set to 100 ms at a ramp rate of 9.42 Hz, resulting in four PASEF ramps with a total cycle time of 0.53 s. Maximum charge was set to 5, and the target intensity was set to 20,000 using an intensity threshold of 2500. Collision energies were set to 20 eV for a 1/K0 of 0.6 V s^−1^ cm^−2^ and to 59 eV for a 1/K0 of 1.6 V s^−1^ cm^−2^ with linear interpolation between these values. Active exclusion was set to 0.4 min.

Acquired MS‐spectra were queried against an in‐house modified TAIR10 database containing protein sequences considering organellar RNA‐editing using the MaxQuant software version 2.6.3.0 (Cox and Mann 2008). Default software settings were applied, and additionally, ‘no fractions’, ‘iBAQ’, and ‘match between runs’ functions were enabled. Afterwards, intensity‐based abundance quantification (iBAQ) values (Schwanhäusser et al. 2011) from the resulting protein groups file were extracted, and contaminants were removed manually. Hierarchical clustering of protein abundance profiles following the Pearson correlation distance function with average linkage was performed using the NOVA software version 0.8.0 (Giese et al. 2015).

### 2.8 NADH‐Oxidation Capacity in Organelle Isolates

Mitochondrial NADH‐oxidation capacity was assessed based on a protocol established for human skeletal muscle (Birch‐Machin et al. 1994) and adapted for isolated I + III_2_ supercomplex from

*Arabidopsis thaliana* (Klusch et al. 2023). In brief, 40 μL of affinity‐purified mitochondria (protein concentration 0.7 μg μl^−1^, solubilised using 5% (w/v) digitonin in solubilization buffer) were used for each assay. The reaction was started by the addition of a buffer containing 25 mM K_2_PO_4_, 5 mM MgCl_2_, 260 μM NADH, 67 μM decylubiquinone, 0.1 mM horse cytochrome *c*, and 3000 U ml^−1^ superoxide dismutase, to a final reaction volume of 300 μL. NADH absorbance was measured at 340 nm in 5 s intervals over a time period of 15 min at room temperature using a plate reader (Multiscan Sky, Thermo Fisher Scientific). To evaluate complex I specific NADH oxidation, enzymatic activity was inhibited with 50 μM rotenone and NADH oxidation was monitored for another 15 min. Mitochondrial protein abundance in each reaction was estimated by submitting small volumes of each sample to semi‐quantitative shotgun proteomics using an Evosep HPLC equipped with an Aurora Elite CSI reverse phase 15 cm C18 column (inner diameter 75 μm, particle size 1.7 μm, pore size 120 Å; IonOpticks), operated in 40 SPD Whisper Zoom mode. Two hundred nanograms of peptide were loaded onto the Evotips according to the manufacturer's instructions. The MS was operated as outlined for shotgun proteomics above. Cumulated iBAQ values of mitochondrial proteins (as deduced from the SUBAcon Hooper et al. 2014) were used to compute relative mitochondrial protein abundance within a sample. The resulting factor was then applied to the protein concentration determined by Bradford to yield absolute mitochondrial protein amounts in each individual assay.

## 3 Results

### 3.1 Cell‐Type‐Specific Mito‐AP Tags Mitochondria in Mesophyll and Guard Cells

Affinity purification of plant mitochondria has become an established technique for isolating small amounts of organelles, particularly for subsequent proteomic analysis. It utilizes ectopic molecular tags fused to outer mitochondrial membrane proteins. Mitochondria carrying the tag are able to bind to magnetic beads coated with a compatible interaction partner. Mitochondria bound to magnetic beads can thus be separated from other, non‐tagged cellular compartments. Mito‐AP requires only a fraction of the plant material and a fraction of the time required for the classical, centrifugation‐based method (Boussardon et al. 2020; Kuhnert et al. 2020; Niehaus et al. 2020). However, potentially the biggest advantage of this technology over conventional methods is the option to express the tag under the control of cell type‐specific promoters and thus provide the means to isolate mitochondria from specific cell types within any given plant organ (Boussardon et al. 2025). The technology, therefore, provides a handle for investigating the functions of specialized mitochondria within selected plant organs.

**FIGURE 1:**
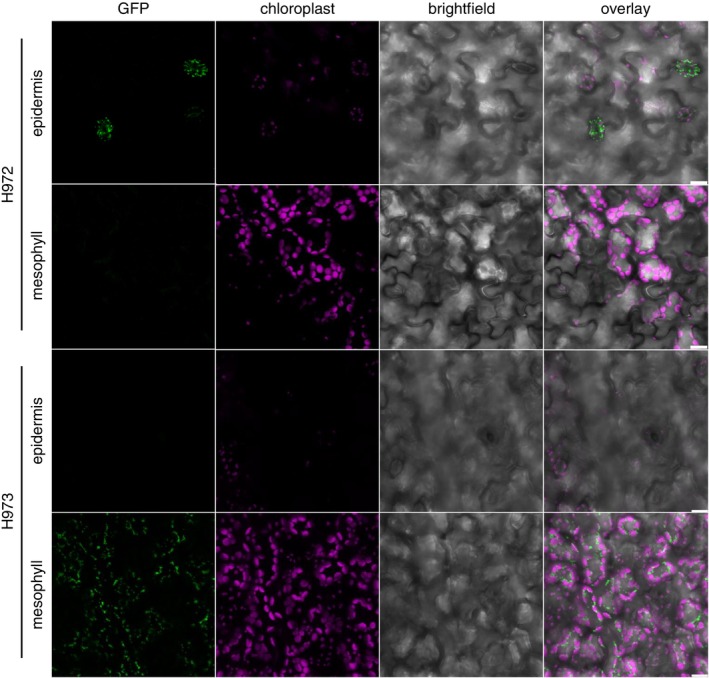
Tagging of mesophyll and guard cell mitochondria by Mito‐AP. Confocal fluorescence microscopy images of leaf sections obtained from lines H972 and H973. From left to right, GFP (green), chloroplast auto‐fluorescence (magenta), brightfield, overlay of all three channels. The top two panels display the same leaf section of a plant stably transformed with the H972 construct, expressing the Mito‐AP fusion protein under the control of a guard cell‐specific promoter. Only selected layers of Z‐stacks, representative of the epidermis or the mesophyll, are shown. The two bottom panels show epidermal and mesophyll focus layers, respectively, of plants stably transformed with the H973 construct aimed at tagging mesophyll cell mitochondria. Scale bars are 10 μm.

Here, two independent *Arabidopsis thaliana* Mito‐AP lines are introduced, which express the fusion protein tag under the control of either a guard cell‐specific or a mesophyll cell‐specific promoter (Yang et al. 2008; Susek et al. 1993). In addition, the original Mito‐AP line (Niehaus et al. 2020) expressing the fusion protein under the control of a 35S promoter (line H500) is used as a control. Since the protein carrying the tag (the ADAPTER) is also fused to a GFP reporter sequence, the specificity of the tagging approach can be tested using confocal fluorescence microscopy. In *Nicotiana benthamiana* leaves that transiently express the H500 Mito‐AP construct, the GFP signal superimposes with the signal obtained from a 35S:*Sc*CoxIV:mCherry construct used as a mitochondrial marker (Niehaus et al. 2020), confirming a mitochondrial localization of the ADAPTER protein. As the construct used here to generate mesophyll and guard cell‐specific lines is the same as in the original publication and varies only in the promoter sequences driving the expression of the tagged protein, the intracellular localization of the fusion protein in the cell type‐specific lines is expected to remain unchanged. Confocal fluorescent images for H972 and H973 leaves (Figure 1) differ considerably from each other, as well as from the H500 line (Figure S1). In H972 plants, discrete GFP fluorescence spots are discernible only in the abaxial epidermis of their leaves. The distribution and shape of the cells displaying GFP signals agree with the tagging of guard cell mitochondria. In contrast, the GFP signal is strong in punctate organelles in mesophyll cells of line H973, and no signal is detectable in the epidermal cell layer or other areas of the leaf. As such, confocal imaging confirms the cell type‐specific expression of the Mito‐AP constructs in the H972 and H973 lines. The two lines are thus deemed suitable for the isolation of mesophyll and guard cell‐specific mitochondria.

### 3.2 Transgenic Expression of the Mito‐AP Tag Does Not Disturb Cellular Metabolism

In order to assess the implications of the ectopic expression of the ADAPTER construct on the cellular metabolism of the Mito‐AP lines, the development of H500, H972, and H973 plants was compared to that of WT plants. Plants carrying either of the three Mito‐AP constructs do not differ in respect to morphology or development from WT plants when grown under long‐day conditions (Figure S2). Neither the transformation process nor the presence of the fusion protein, therefore, has noticeable effects on the main metabolic functions.

To identify possible implications caused by the expression of the Mito‐AP constructs on the molecular level, leaf cell protein extracts of H972, H973, and H500 plants were compared to those of WT plants by liquid chromatography coupled to tandem mass spectrometry (LC–MS/MS). Using four biological replicates for each line, principal component analysis (PCA) results in a single broad cluster containing all 16 samples, thus indicating a lack of individual features across the four sample groups (Figure S3A). This is further corroborated by pairwise comparisons of each of the three Mito‐AP lines with the WT. Except for the ADAPTER protein (AT1G55450), not a single protein group differs from the WT in either of the Mito‐AP lines within the chosen significance parameters (FDR ≤ 0.05, S0 ≥ 0.1; Figure S3B). Likewise, the ADAPTER protein is also the only significant difference in the comparison between H972 and H973 (Figure S3B). Its higher abundance in H973 indicates that the mesophyll‐specific promoter drives stronger expression of the tag compared to the guard cell‐specific promoter. It can thus be concluded that (1) the presence of the tagged ADAPTER protein has no or only minor impact on global protein abundance; (2) the metabolism of all Mito‐AP lines resembles that of the wild type; and (3) potential differences between the mitochondrial proteomes of H972 and H973 reflect their respective functional requirements and cellular environments, and are not caused by the ectopic expression of the Mito‐AP construct.

### 3.3 Organelle Isolations From Leaves of Transgenic Plants by Mito‐AP Yield Highly Enriched Mitochondrial Fractions

Leaves harvested from H972, H973, and H500 plants 2 h into the light period were submitted to Mito‐AP, followed by LC–MS/MS of the respective isolates. Comparison of these results to the LC–MS/MS data obtained from crude cellular extracts of the three Mito‐AP lines allows assessment of organelle enrichment by cell type‐specific Mito‐AP. For this, subcellular localisations for all identified protein groups were assigned by the SUBAcon algorithm (Hooper et al. 2014), and relative protein abundance of each cell compartment was estimated using cumulated iBAQ values. However, due to the use of intact leaves containing all cell types (rather than mesophyll cells and guard cells only, which may differ in the mitochondrial contribution towards total cellular protein content), the enrichment factors provided by this approach should be considered as rough estimates. Still, they provide valuable reference points to judge the overall success of the mitochondria isolation procedure.

**FIGURE 2:**
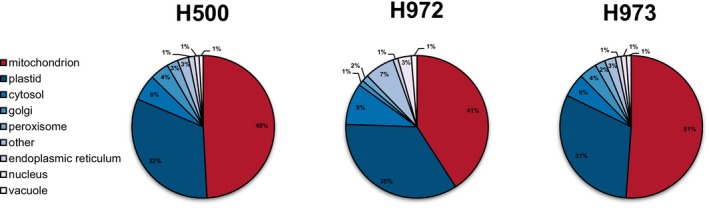
Median distribution of protein abundance across different subcellular compartments in Mito‐AP isolates. Mitochondrial affinity purification obtained from the three Mito‐AP lines H500 (tagging of mitochondria under control of a 35S promoter), H972 (tagging of mitochondria under control of a guard cell‐specific promoter), and H973 (tagging of mitochondria under control of a mesophyll cell‐specific promoter) were subjected to LC–MS/MS analysis. Assignment of protein groups to subcellular compartments was then performed using the SUBAcon algorithm (Hooper et al. 2014). Cumulated median iBAQ values for each subcellular compartment were subsequently calculated using four independent biological replicates and are provided as relative amounts. Mitochondrial protein abundance is displayed in dark red, chloroplast protein abundance in dark blue, minor contaminants are displayed in shades of light blue.

Abundance of cytosolic and plastid proteins in mitochondrial isolates is reduced by ~50% compared to the crude cellular extracts (Figures 2 and S4). In contrast, the relative abundance of mitochondrial proteins increases from an average of ~5% in the crude leaf extracts to an average of ~50% in the mitochondrial isolates. Mitochondria enrichment is thus in line with values reported previously for this Mito‐AP protocol (Niehaus et al. 2020). However, particularly for the guard cell‐specific H972 line, organelle enrichment is subject to considerable variation (Figure S4B,C). Reasons for this are currently unknown, but given that the mitochondrial content in the leaf extracts of this line does not show a similar degree of variation (Figure S4A), we speculate that conditions during isolation are more critical for this line and have a stronger influence on the outcome of the procedure.

**FIGURE 3:**
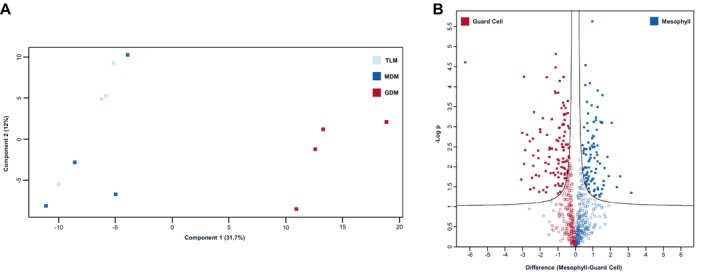
Statistical appraisal of differing mitochondrial protein abundance in guard cells and mesophyll cells. Total leaf mitochondria (TLM), mesophyll‐derived mitochondria (MDM) and guard cell‐derived mitochondria (GDM) were isolated by Mito‐AP in four biological replicates and subjected to shotgun proteomics. Protein groups were subsequently assigned to their subcellular compartments using the SUBAcon algorithm. Mitochondrial protein abundance was normalised using the width adjustment function of the Perseus software package. (A) PCA of mitochondrial protein group abundance of TLM (light blue), MDM (dark blue) and GDM (red). (B) comparison of log_2_‐transformed LFQ‐values of GDM and MDM protein groups. Volcano plot y‐axis, negative logarithmic (to the base of 10) *p*‐value; x‐axis, difference between MDM and GDM of log_2_‐transformed LFQ‐values. Protein groups located in the areas above the black lines fulfil the significance threshold limits (FDR ≤ 0.05; S0 ≥ 0.1). Protein groups depicted in red are of higher abundance in guard cell mitochondria, protein groups shown in blue are of higher abundance in mesophyll cell mitochondria.

Nevertheless, the enrichment factor is > 6 for all replicates (Figure S4C). Considering that (1) the tag used for the organelle pull‐down is targeting mitochondria (Niehaus et al. 2020), (2) the expression of the Twin‐Strep‐tag:GFP:ADAPTER constructs is cell type‐specific (Figure 1), and (3) pull‐downs using these constructs yield enriched mitochondrial fractions (Figures 2 and S4C), it seems justified that the products of the organelle pull‐downs of lines H972 and H973 are referred to as guard cell‐derived mitochondria (GDM) and mesophyll cell‐derived mitochondria (MDM), respectively, from here on. Mitochondria enriched from the H500 Mito‐AP line will be referred to as total leaf mitochondria (TLM).

### 3.4 Mesophyll Cell and Guard Cell Mitochondria Proteomes Differ Distinctly

Using protein abundance as a proxy, comparative proteomics of samples taken under steady‐state conditions provides valuable information on the differing physiological properties of the samples. Across the three sample groups (GDM, MDM, TLM), detection of mitochondrial protein groups is remarkably similar (Figure S5). Among the 866 mitochondrial protein groups found during the course of this study (Table S1), 802 are shared between GDM, MDM, and TLM (Figure S5, Table S2). Merely two protein groups were detected exclusively in GDM (AT2G45330, AT1G16740), whereas three protein groups are limited to MDM (AT3G51090, AT3G25430, AT1G02020) and TLM (AT5G03560, AT1G74260, AT1G48570), respectively (Table S2). MDM and TLM share 43 proteins not detected in GDM, thus emphasizing the similarities between the two sample groups (Figure S5, Table S2). These findings also largely conform with similar numbers for cell type‐exclusive detection as reported by Hater et al. (this issue).

Since the varying degrees of organellar contamination (Figure S4) will inevitably interfere with the statistical appraisal of mitochondrial protein abundance, non‐mitochondrial accessions were excluded from normalization of protein abundance and subsequent statistical testing. The reduction of the dimensionality of all three sample groups by PCA (Figure 3A) reveals two distinct clusters. The first of these clusters consists of members of the TLM and MDM sample groups. Considering that mesophyll cells are the major contributors toward the TLM pool, co‐clustering of these sample groups is not surprising. In contrast, members of the GDM sample group cluster separately from the MDM and TLM samples. The PCA thus indicates distinctive mitochondrial proteomes among leaf cells. This notion is confirmed by 205 protein groups differing significantly in abundance between GDM and MDM (Figure 3B; Table S3).

### 3.5 A Different Mode of NADH Production in Guard Cell Mitochondria

**FIGURE 4:**
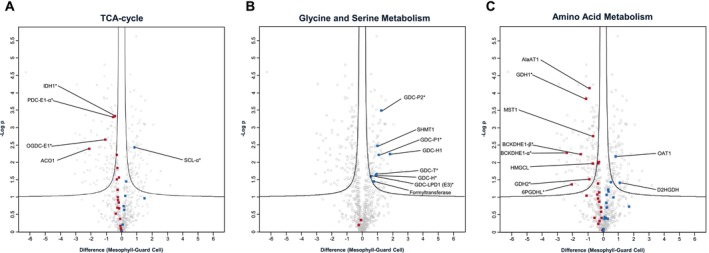
Abundance differences between protein groups of major NADH‐producing pathways in guard cell and mesophyll cell mitochondria. After normalization, log_2_‐transformed LFQ values of protein groups assigned to mitochondria by the SUBAcon algorithm were used to assess differences in the mitochondrial proteomes of guard cells and mesophyll cells by statistical testing employing four independent biological replicates. Volcano plot x‐axis, difference between log_2_‐transformed LFQ‐values; y‐axis, −log *p*‐values. Proteins located above the black lines fulfil the selection criteria for a significantly different abundance (FDR ≤ 0.05; S0 ≥ 0.1). Protein groups displayed in dark red are of higher abundance in GDM, protein groups shown in blue are of higher abundance in MDM. Enzymes producing NADH are marked by an asterisk.

The main sources of reductive power ultimately driving oxidative phosphorylation in leaf mitochondria are the TCA cycle, the mitochondria‐located steps of PR, and general amino acid catabolism. For the TCA cycle, closely associated enzymes, such as the subunits of the pyruvate dehydrogenase complex (PDC) and malic enzyme (ME) isoforms, are also included in this analysis, although they are not pathway‐defining enzymes. In general, protein groups of the extended TCA cycle pathway show a tendency towards an increased abundance in GDM (Figure 4A). Significantly increased abundance of subunits of the PDC and the oxoglutarate dehydrogenase complex (OGDC) is recorded, albeit these being less than two times higher in GDM. The most conspicuous difference is observed for aconitase 1 (ACO1), which catalyzes the conversion of citrate to isocitrate and is over four times more abundant in GDM. Interestingly, one of the regulatory subunits of the enzyme catalyzing the following reaction, the conversion of isocitrate to 2‐oxoglutarate by the isocitrate dehydrogenase (IDH), is also of increased abundance in GDM (IDH1; AT4G35260). The only exception to the general trend of TCA cycle protein groups is the succinate CoA‐ligase (SCL) alpha‐isoform AT5G23250, which is of significantly lower abundance in GDM. However, its other isoform (AT5G08300) is of slightly higher abundance in GDM, although this is not statistically confirmed (*p*‐value > 0.1). Since AT5G08300 is the dominant isoform, being > 10 times more abundant than AT5G23250 (based on iBAQ values), the decreased amount of AT5G23250 in GDM is expected to have little bearing on the overall SCL activity. Similar abundance differences of SCL isoforms in GDM and MDM were also detected by Hater et al. (this issue). Together, these data suggest an increased capacity for pyruvate oxidation in GDM.

Interestingly, fumarase 2 (fum2) is showing the second‐highest abundance increase in GDM, a result which is also corroborated by Hater et al. (this issue). Although fluorescent fusion protein analysis suggests a cytosolic origin (Pracharoenwattana et al. 2010), the protein was repeatedly identified in isolated mitochondria (Millar et al. 2001; Heazlewood et al. 2004; Finkemeier et al. 2011; Taylor et al. 2011; König et al. 2014; Nietzel et al. 2020). It is among our list of mitochondrial proteins since SUBAcon states both cellular compartments for this protein. Its enrichment in mitochondria isolates indicates considerable interactions between the enzyme and the organelle. Malate‐producing mitochondrial fumarase 1 (fum1) and fum2 are both of increased abundance in GDM (fum1 by > 1.2 times, *p*‐value 0.014; fum2 by > 8 times, *p*‐value 0.021). Since malate plays a crucial role in the regulation of stomatal conductance, it can be speculated that fum2 has functions related to this process. Its (potential) association with mitochondria, however, cannot be explained by our data and awaits further attention. In any case, mitochondrial enzymes using malate as their substrate, the mitochondrial malate dehydrogenase (mMDH1, mMDH2) and NAD‐dependent malic enzyme (NAD‐ME1, NAD‐ME2) do not differ significantly in abundance. Hence, an altered cytosolic malate metabolism in guard cells does not seem to radiate into the mitochondrial compartment.

In contrast to TCA‐cycle related enzymes, the mitochondria‐located components of the PR pathway are less abundant in GDM (Figure 4B). This includes subunits H, P, LPD1, and T of the GDC, a serine hydroxymethyltransferase (SHMT1), as well as a 10‐formyl tetrahydrofolate (THF) deformylase (AT4G17360), whose activity prevents excessive accumulation of THF and is therefore essential for leaf PR capacity (Collakova et al. 2008). The same 10‐formyl THF isoform was also found to be less abundant in GDM by Hater et al. (this issue). Notably, Hater et al. found another THF deformylase homolog (AT5G47435) to be of higher abundance in guard cells. In our study, AT5G47435 shows the same trend, albeit in a non‐significant fashion. The data therefore suggest that the two THF deformylase homologs AT5G47435 and AT4G17360 are regulated differently in response to altered PR activity and that AT5G47435 may serve guard cell‐specific functions not related to PR. In summary, the observed differences in the abundance of PR‐related enzymes clearly suggest a marked decrease, but not a cessation, of PR capacity in GDM.

Mitochondrial enzymes involved in the metabolism of amino acids other than glycine and serine are mostly of higher abundance in guard cells (Figure 4C). Catabolism of these amino acids ultimately produces TCA‐cycle intermediates and may thus enhance NADH production in GDM. In particular, enzymes involved in alanine (Ala), glutamate (Glu), and branched‐chain amino acid (BCAA) degradation are noteworthy. Metabolic flux models indicate an increased Glu synthesis in guard cell chloroplasts. Malate oxidation yields the NADPH required for this, and Glu is subsequently exported via the dicarboxylate transporter into the cytosol (Robaina‐Estévez et al. 2017). However, known mitochondrial transporters of this amino acid (such as UCP1, UCP2, UCP3, BOU; (Monné et al. 2018; Porcelli et al. 2018)) are either of similar abundance (UCP1, AT3G54110), were not found (UCP2, AT5G58970), or are of lower abundance in GDM (UCP3, AT1G14140; BOU, AT5G46800, Figure S6), suggesting a lower transport capacity for this amino acid into GDM. Instead, Glu may be produced within guard cell mitochondria themselves. Using Ala as the amino‐group donor, alanine aminotransferase 1 (AlaAT1, AT1G17290) produces pyruvate and Glu from Ala and 2‐OG. AlaAT1 is indeed of higher abundance in GDM, but the origin of Ala in these organelles is unknown. However, in the presence of Ala and without any further import of Glu, the combined action of AlaAT and GDH within a 2‐OG centered cycle could produce reduction equivalents (in the form of NADH) for respiration as well as pyruvate for the TCA cycle. Within the mitochondrial compartment, alanine oxidation may therefore be more efficient than pyruvate oxidation alone.

Nevertheless, Glu may also result from the degradation of BCAAs (leucine, isoleucine, and valine). After an initial transamination of 2‐OG, yielding Glu and α‐keto acids, the latter are further oxidized by the NAD‐dependent activity of the branched chain keto‐acid dehydrogenase (BCKDH) complex in mitochondria. Apart from the production of NADH, this step also produces acetyl‐CoA, which enters the TCA cycle to yield additional reduction equivalents for ATP synthesis. The two E1‐subunits of the BCKDH complex, BCKDHE1‐α and BCKDHE1‐β, are of significantly higher abundance in GDM, but evidence for an increased oxidation of BCAAs in GDM is patchy, since the mitochondria‐located branched chain amino transferase (AT1G10060) located upstream of BCKDH was not detected, nor does the E2‐subunit of the complex (AT3G06850) show any differential abundance. In contrast, Hater et al. (this issue) find the E subunit (bce2) to be of higher abundance in guard cells. Two other enzymes involved in BCAA catabolism are of significantly higher abundance in GDM in our study. Acetyl CoA‐producing hydroxymethylglutaryl‐CoA lyase (HMGCL; AT2G26800) and NADH‐producing 3‐hydroxyisobutyrate dehydrogenase (putative 3‐hydroxyisobutyrate dehydrogenase/6PGDHL, AT4G29120) are involved in leucine and valine degradation, respectively, downstream of BCKDH. Overall, 12 of the 18 protein groups involved in BCAA degradation identified in this study are of higher abundance in GDM (four significantly, eight non‐significantly). The only member of this group with a significantly lower abundance in GDM is a dihydrolipoamide dehydrogenase (LPD1; AT1G48030). Since it not only participates in the BCKDH complex but also in PDC, OGDC, and GDC, its differential abundance may result from participation in these other complexes. Besides Glu, Ala, and BCAA degradation, few other protein groups involved in amino acid metabolism are of significantly higher abundance in GDM. The mercaptopyruvate sulfurtransferase MST1 (AT1G79230) catalyzes the conversion of 3‐mercaptopyruvate to pyruvate and hydrogen sulfide (H_2_S) using reduced thioredoxin as an electron donor. While pyruvate may fuel PDC activity and ultimately also the TCA cycle to produce reduction equivalents for oxidative phosphorylation (OXPHOS), an increased enzymatic capacity of MST1 could result in increased H_2_S‐production. As a signaling molecule, H_2_S can convey protection from stress by inducing ROS protection mechanisms (Corpas and Palma 2020). MST1 is also reported to be more abundant in GDM by Hater et al. (this issue).

Besides LPD1 (which is part of several protein complexes with different activities), only two protein groups involved in amino acid metabolism are of lower abundance in GDM. D‐2‐hydroxyglutarate dehydrogenase (D2HGDH; AT4G36400) is part of the lysine catabolic pathway and catalyzes the oxidation of D‐2‐hydroxyglutarate to 2‐OG. This reaction provides electrons for the ubiquinone pool via the electron transfer flavoprotein/electron transfer flavoprotein ubiquinone oxidoreductase (ETF/ETFQOR) pathway (Araújo et al. 2010). The second protein group with lower abundance in GDM is the ornithine delta‐aminotransferase 1 (OAT1, AT5G46180). It is involved in arginine degradation downstream of arginase. Arginase 1 (ARGAH1, AT4G08900) is of similar abundance in MDM and GDM, but arginase 2 (ARGAH2, AT4G08870) is of higher abundance in GDM, although this difference is statistically not significant (*p*‐value 0.084). Boussardon et al. (this issue) detected a higher abundance of ARGAH1, ARGAH2, and OAT1 in guard cell mitochondria when compared to their mesophyll cell counterparts. The authors propose that an increased arginine and proline metabolism contributes to the production of reducing equivalents, thus supporting ATP synthesis in guard cells.

In summary, the breakdown of amino acids produces reduction equivalents in the form of NADH and FADH_2_, as well as intermediates which are eventually funneled into the TCA cycle. The observed increase in TCA cycle enzyme abundance can therefore (at least in part) be attributed to an increased provision of carbon skeletons derived from amino acid degradation. Guard cell mitochondria, therefore, seem to utilize amino acids other than glycine to compensate for the lack of PR‐related NADH production for ATP synthesis. While being beyond the scope of this study, it would nevertheless be interesting to test if the use of non‐glycine amino acids for energy conversion also pulsates over the diurnal cycle in the same way it is reported for glycine (Lee et al. 2010).

**FIGURE 5:**
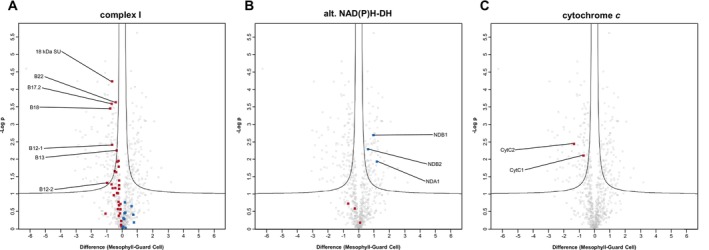
Protein abundance differences between protein groups of selected respiratory enzymes in guard cell and mesophyll cell mitochondria. After normalization, log_2_‐transformed LFQ values of protein groups assigned to mitochondria by the SUBAcon algorithm were used to assess differences in the mitochondrial proteomes of guard cells and mesophyll cells by statistical testing employing four independent biological replicates. Volcano plot x‐axis, difference between log_2_‐transformed LFQ‐values; y‐axis, −log *p*‐values. Proteins located above the black lines fulfil the selection criteria for a significantly different abundance (FDR ≤ 0.05; S0 ≥ 0.1). Protein groups displayed in dark red are of higher abundance in GDM, protein groups shown in blue are of higher abundance in MDM.

In any case, reduction equivalents produced by the TCA cycle, PR, and the oxidation of non‐PR amino acids are re‐oxidized by complex I and by the alternative NAD(P)H dehydrogenases of the plant OXPHOS system. Complex I (CI) subunits show a clear bias towards the GDM site of the volcano plot, and a total of seven proteins are of significantly higher abundance in GDM (Figure 5A). In contrast, all of the identified non‐proton pumping alternative NAD(P)H dehydrogenases (the two external dehydrogenases NDB1 and NDB2, as well as the internal dehydrogenase NDA1) are of lower abundance in GDM (Figure 5B).

Abundance of respiratory chain complexes II, III, and IV (CII, CIII, and CIV) is very similar between GDM and MDM, with only two significant abundance changes for CII, one for CIII, as well as for CIV, and three for CV (Figure S7). The residual subunits distribute fairly evenly between the GDM and MDM sides of the volcano plots, suggesting no differences in complex abundance. However, the two isoforms of the mobile electron carrier cytochrome *c* are of significantly increased abundance in GDM (Figure 5C), whereas the single AOX isoform detected here (AOX1A, AT3G22370) is non‐significantly decreased in GDM (*p*‐value 0.08, Figure S7). Altogether, the data suggest that more electrons enter the respiratory chain at complex I in GDM and that proton‐pumping components of the respiratory chain are preferred, while alternative, non‐proton‐pumping enzymes play a lesser role. The abundance of the ATP‐synthase subunits is comparable between the two compartments (Figure S7). The overall capacity for ADP‐phosphorylation is therefore expected to be similar in GDM and MDM.

### 3.6 Complex I Interaction Patterns in GDM Largely Resemble Those of MDM and TLM

**FIGURE 6:**
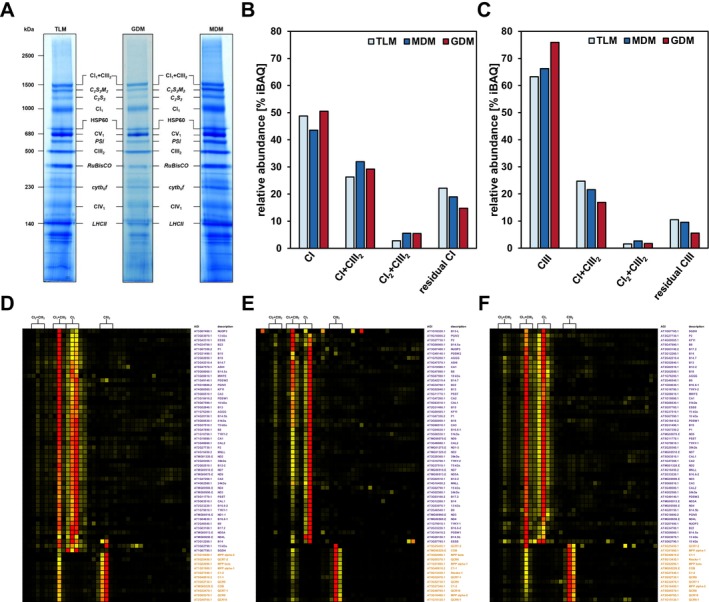
BN‐PAGE, prot and protein abundance profiles of complex I and III subunits of Mito‐AP isolates. Following Mito‐AP, bead‐bound TLM, GDM, and MDM were digitonin‐solubilised, and 185 μg protein were subsequently loaded onto a BN‐gel. After separation, protein complexes were stained with Coomassie (A). TLM, GDM, and MDM gel lanes were cut into 39 fractions, each of which was then subjected to tryptic in‐gel digestion and LC–MS/MS analysis. Protein abundance at the positions of singular complex I, dimeric complex III, as well as at the positions of supercomplexes I_1_ + III_2_ and I_2_ + III_2_ profiles were created from iBAQ values (B, C). D to F, hierarchically clustered protein abundance profiles of complex I and complex III subunits of TLM (D), GDM (E) and MDM (F). Highest relative protein abundance is depicted in red, medium abundance in orange, and low abundance in yellow. Black colour indicates a lack of detection. Arabidopsis gene identifier (AGI) and protein descriptions are given to the right of the heatmaps. Purple IDs indicate complex I subunits, orange IDs those of complex III. Brackets on top of the heatmaps indicate fractions for which abundance values were cumulated to calculate complex/supercomplex abundance relative to total mitochondrial protein content. Complete heatmaps are available at: https://complexomemap.de/projects‐guard/.

Respiratory complexes I and III interact with each other by forming respiratory supercomplexes (Eubel et al. 2003; Dudkina et al. 2005; Klusch et al. 2023; Waltz et al. 2024). In order to test if the increased CI+++ subunit abundance in GDM also affects the interaction of this enzyme complex with complex III, digitonin‐solubilised GDM, MDM, and TLM were submitted to blue‐native polyacrylamide gel electrophoresis (BN‐PAGE). In concordance with the shotgun LC–MS/MS results, mitochondrial isolates of all three lines are contaminated by chloroplast proteins, hence showing banding patterns originating from both organelles (Figure 6A) (Eubel et al. 2003; Schröder et al. 2022). After staining, the BN gel lanes were cut into 39 fractions each, which were then submitted to tryptic in‐gel digestion. Peptides were extracted from the gel matrix and subsequently analysed by LC–MS/MS. In total, 503, 596, and 636 mitochondrial protein groups were detected for GDM, MDM, and TLM, respectively. Protein abundance profiles across all 39 gel fractions were produced, in which iBAQ values of each single fraction were normalized against the total amount of that protein group. Distribution of CI and CIII subunits indicates the presence of individual complexes CI_1_ and CIII_2_, as well as two CI/CIII supercomplexes, CI_1_ + CIII_2_ and CI_2_ + CIII_2_ (Figure 6D–F), which conforms with previously published results (Eubel et al. 2003). Relative cumulated iBAQ values show a similar distribution of CI and CIII between the individual complexes and their supercomplexes in all three lines (Figure 6B,C). GDM, MDM, and TLM thus do not seem to differ in their CI/CIII interaction pattern. However, the amount of CI and CIII subunits found outside the major complex/supercomplex bands is slightly lower in GDM (Figure 6B,C), suggesting lower amounts of assembly/breakdown products in guard cell mitochondria.

### 3.7 NADH Re‐Oxidation Capacity Differs Between GDM and MDM/TLM

**FIGURE 7:**
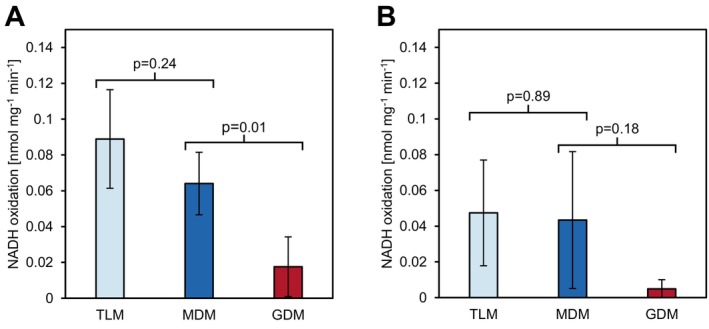
NADH oxidation rates in TLM, GDM, and MDM. Four replicates of Mito‐AP isolated TLM, MDM, and GDM were submitted to digitonin‐solubilization. 28 μg of protein were then mixed with a buffer containing K_2_PO_4_, MgCl_2_, NADH, decylubiquinone, cytochrome *c* and superoxide dismutase, before the decrease of NADH absorption was followed at 340 nm over a period of 15 min before (A) or after (B) addition of 50 μM rotenone. To estimate the relative contribution of mitochondrial protein to the total protein content of the isolates, aliquots of the same isolation were submitted to LC–MS/MS analysis. Cumulated iBAQ values of all mitochondrial proteins were divided by total iBAQ intensity and the resulting factors were applied to the protein concentrations as determined by Bradford.

The somewhat mixed results on CI obtained by shotgun LC–MS/MS and by BN gel analysis raise the question of the enzymatic capacity of this important point of entry for electrons into the respiratory chain in guard cell mitochondria. In order to assess NADH‐reduction capacity and electron transfer efficiency, the enzymatic capacity to oxidize NADH and the transfer of electrons onto cytochrome *c* were quantified in the presence of decylubiquinone and cytochrome *c* for GDM, MDM, and TLM (Birch‐Machin et al. 1994; Klusch et al. 2023). Since the degree of organelle enrichment varies between the isolates, which will inevitably affect the abundance and enzymatic capacity of NADH‐oxidizing enzymes, normalization of assay results was deemed necessary. To this end, ratios of mitochondrial protein abundance over total protein abundance were determined for aliquots of each of the isolates used for the NADH‐oxidation assay by LC–MS/MS analysis (Table S4). These ratios were then applied to Bradford‐determined total protein concentrations, yielding absolute mitochondrial protein abundance for each sample. TLM show the highest NADH‐oxidation rates, followed closely by MDM and, with a much more pronounced gap, by GDM (Figure 7A). The data therefore indicate that the total NADH oxidation capacity is reduced in GDM when compared to MDM. In order to distinguish between complex I capacity on one hand and that of the alternative NAD(P)H dehydrogenases on the other hand, assays were repeated in the presence of the complex I inhibitor rotenone (Figure 7B). Rotenone‐insensitive NADH‐oxidation clearly contributes massively towards the observed NADH‐oxidation rates. Despite our best efforts, variance in the presence and in the absence of rotenone is high for all lines, making it difficult to calculate complex I catalyzed NADH‐oxidation capacity. However, another parameter to judge mitochondrial ATP‐production is the capacity of organelles to transport ATP, ADP, and inorganic phosphate across the inner membrane. GDM and MDM demonstrate clear differences in components involved in these transport processes, nearly all of which are of lower abundance in GDM (Table S3; Figure S6). This includes isoforms of ADP/ATP carriers (AAC1, non‐significantly; AAC3, significantly), the mitochondrial phosphate transporters (MPT2, significantly; MPT3, non‐significantly), mitochondrial ATP‐MgPi transporters (APC1, significantly; APC3, non‐significantly), and the adenine nucleotide transporter 1 (ADNT1, non‐significantly). Merely AAC2 is of slightly higher abundance in GDM (non‐significantly). Therefore, the contribution of each individual guard cell mitochondrion towards the fulfillment of cellular ATP demand is expected to be lower than it may be the case for its MDM counterparts.

### 3.8 Regulation of Protein Function

Three protein groups (MKK2, PP2A‐B′‐zeta, and ABC1K10A) directly regulating enzymatic functions are of higher abundance in GDM (Figure S8A), potentially showcasing the need for tighter regulation of mitochondrial functions in guard cells. MKK2 (AT4G29810.2) is a mitogen‐activated protein kinase (MAPK) kinase. As part of a red light‐dependent phosphorylation cascade, it works downstream of phytochrome B (PhyB) and upstream of MAP kinase homolog 2 (MPK2) to trigger stomatal opening (Li et al. 2023). PP2A‐B′‐zeta (AT3G21650) is a regulatory B′ subunit of the serine/threonine‐specific protein phosphatase 2A (PP2A). It localizes to the cytosol as well as to mitochondria (Matre et al. 2009). PP2A‐type phosphatases are involved in regulating the phosphorylation status of SPEECHLESS (SPCH), a transcription factor involved in regulating stomatal development (Bian et al. 2020), but its functions within the mitochondrial environment are currently unknown. ABC1K10A (AT1G1139) is an atypical kinase, which is mitochondria‐located and involved in preventing ROS accumulation under salt and osmotic stress conditions (Qin et al. 2020). Altogether, these results suggest that particularly phosphorylation/dephosphorylation events play important roles in regulating the activities of mitochondrial proteins within guard cells.

### 3.9 Assembly and Integrity of OXPHOS Complexes

The assembly of OXPHOS complexes relies on proteins that temporarily interact with nascent complexes but are not part of the mature complex. A total of 14 of these assembly proteins were detected in LC–MS/MS analysis of GDM and MDM, the majority of which are involved in CIV assembly. Six assembly and maintenance proteins are significantly more abundant in GDM, two of which are involved in CIV assembly (Cox11, Cox15; Figure S8B, Table S3), whereas another CIV assembly protein (HCC2) is of higher abundance in MDM. Another group consists of two AFG1‐like proteins, which accumulate in GDM. Human AFG1 (LACE1) is involved in the protein homeostasis of nuclear‐encoded complex IV subunits, as well as maintaining complex III and complex IV activity (Cesnekova et al. 2016), and a similar role is suggested for yeast AFG1 (Khalimonchuk et al. 2007). All three AFG1‐like proteins detected during the course of this study (AFG1‐like1, AT2G25530; AFG1‐like2, AT4G28070; AFG1‐like3, AT4G30490; TAIR lists all these accessions as AFG1‐like, the consecutive numbering is introduced here for the sake of simplicity) are of higher abundance in GDM (non‐significantly for AFG1‐like2). AFG1‐like1 is 6.2‐fold (73.5 times) more abundant in guard cell mitochondria, which makes it the protein group showing the highest overall difference. However, despite this strong concentration within GDM, it cannot be considered a guard cell‐specific protein, since it was also detected in MDM, TLM, as well as Arabidopsis cell culture mitochondria (Nelson et al. 2013; Fuchs et al. 2020; Rugen et al. 2019). AFG1‐like2 and AFG1‐like3 also seem to localize to mitochondria (although not exclusively in the case of AFG1‐like3) (Bussemer et al. 2009). Taken together, a clear tendency towards the assembly or protection of respiratory chain components, specifically of CIV, is observed for GDM.

### 3.10 Proteome Maintenance in Guard Cell Mitochondria

**FIGURE 8:**
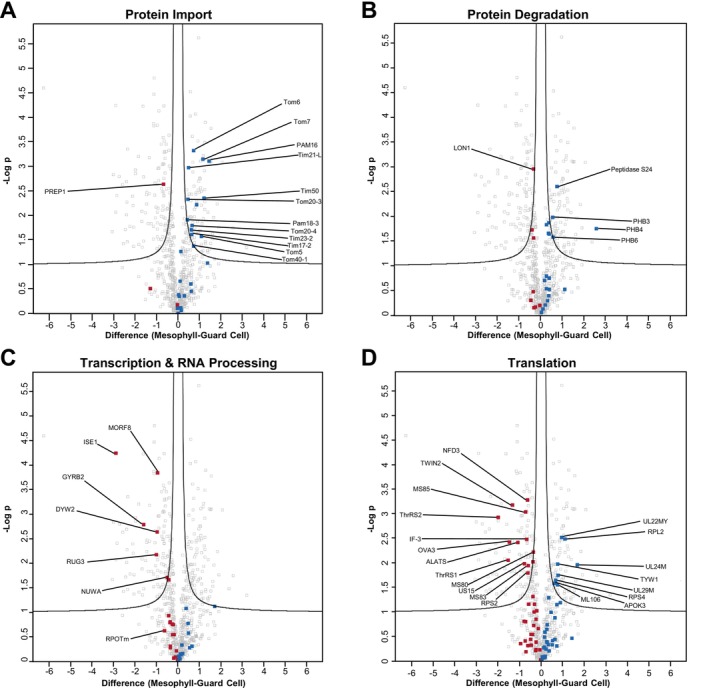
Protein abundance differences between protein groups of proteome maintenance processes in guard cell and mesophyll cell mitochondria. After normalization, log_2_‐transformed LFQ values of protein groups assigned to mitochondria by the SUBAcon algorithm were used to assess differences in the mitochondrial proteomes of guard cells and mesophyll cells by statistical testing employing four independent biological replicates. Volcano plot x‐axis, difference between log_2_‐transformed LFQ‐values; y‐axis, −log *p*‐values. Proteins located above the black lines fulfil the selection criteria for a significantly different abundance (FDR ≤ 0.05; S0 ≥ 0.1). Protein groups displayed in dark red are of higher abundance in GDM, protein groups shown in blue are of higher abundance in MDM.

Differing physiological roles of guard cell and mesophyll cell mitochondria may put differing strains on organelle proteins. Hence, proteome maintenance measures for preserving organelle functions may differ between these cell types. At first glance, the processes influencing mitochondrial protein homeostasis reveal conflicting tendencies. The majority of protein import components are of lower abundance in GDM (as is also independently reported by Hater et al., this issue), thus strongly suggesting a reduced capacity of guard cell mitochondria for taking up nuclear‐encoded proteins (Figure 8A). Protein groups involved in protein degradation, such as the mitochondria‐located DegP10 protease (AT5G36950), as well as members of the CLP and FTSH families, are largely inconspicuous, showing only non‐significant and minor abundance differences. Three proteins of the prohibitin family (PHB3, 4, 6), however, are of lower abundance in GDM (Figure 8B). A stabilizing function of these proteins was reported for the FTSH protease. Together, FTSH and PHB subunits form a ~2 MDa complex (Piechota et al. 2010; Steglich et al. 1999; Joshi et al. 2025). Although the main abundance of PHBs migrates at 1 MDa in BN‐PAGE, other peaks of PHB abundance between 1.5 and 3.0 MDa coincide with peaks of FTSH subunits in GDM and MDM, albeit at lower abundance in GDM (Figure S9). Interestingly, another protein involved in mitochondrial protein degradation, peptidase S24, shares high‐molecular mass abundance peaks with FTSH subunits and PHBs. In GDM, lower abundance of peptidase S24 across the entire mass region of the BN gel (Figure S9) matches significantly lower abundance of this protein in our LC–MS/MS shotgun data (Table S3, Figure 8B). The role of this enzyme in mitochondrial protein homeostasis is currently unknown, since its knockout has no discernible effect on mitochondria (Migdal et al. 2017), but these results suggest physical interactions of FTSH with peptidase S24 and PHBs in a cell type‐specific manner.

In contrast, LON1, a double‐function chaperone/protease (Li et al. 2017) is of higher abundance in GDM. Knock‐out mutants of this protein show distinctly differing protein degradation patterns when compared to WT, particularly in respect to degradation of HSP70, prohibitins, and mitochondria‐localised enzymes of the photorespiratory pathway. Except for a single HSP70 (AT3G09440.2), all other reported LON1 targets are of lower abundance in GDM, thus supporting a role in the regulation of mitochondrial functions for this protease.

Components involved in organellar protein synthesis seem to be of higher abundance in GDM (Figure 8C,D), particularly those involved in transcription and RNA processing. Increased size exclusion limit 1 (ISE1, AT1G12770) encodes a mitochondrial DEAD‐box RNA helicase. Knock‐out mutants of ISE1 produce higher amounts of hydrogen peroxide than their wild‐type counterparts and seem to possess a reduced inner membrane potential. *ise1* mitochondria are hence expected to possess impaired transport of electrons along the proton‐pumping components of the respiratory chain (Stonebloom et al. 2009). For several other proteins, such as MORF8, DYW2, and NUWA, which are also of higher abundance in GDM (Figure 8C), the situation is more complex. These essential components of the organellar RNA‐editing machinery are dual‐targeted to mitochondria and chloroplasts. Since GDM as well as MDM fractions are contaminated with chloroplasts, the increased abundance of these proteins in GDM may result from the presence of chloroplasts in the isolated mitochondrial fractions. However, these proteins decrease drastically in the mitochondrial guard cell proteome when compared to the mitochondrial proteomes of mesophyll and vascular cells under carbon starvation conditions (Boussardon et al., this issue). Components related to organellar translation also show a tendency towards higher abundance in GDM (Figure 8D). While ribosomal subunits show no or only little bias towards GDM, tRNA‐synthases such as OVA3, TWIN2, ALATS, and ThrRS2 clearly tend to be more abundant in GDM, but most of these proteins (OVA3, ALATS, ThrRS2) are dual‐targeted to mitochondria and chloroplasts/cytosol. Similar to the situation described here for the editing factors, it is therefore difficult to tell if the higher protein group abundance in GDM is indeed due to increased mitochondrial abundance.

Taken together, protein groups involved in mitochondrial proteome maintenance indicate a strong tendency for lower protein import capacity in GDM, which does not align well with the somewhat ambivalent data on organellar protein synthesis. Protein degradation potentially also exerts an influence on maintaining cell‐type‐specific mitochondrial proteomes, as showcased here by different abundances/BN‐migration patterns of LON1, FTSH, and S24 peptidase.

## 4 Discussion

### 4.1 Mito‐AP Enables Cell Type‐Specific Isolation of Leaf Mitochondria

Up to now, leaf mitochondrial isolates were almost exclusively bulk fractions containing mitochondria from different cell types, including mesophyll, vascular tissue, and epidermal tissue. It is therefore currently unknown if and how mitochondria from distinct leaf cell types differ within this organ. Targeted, cell type‐specific isolation of mitochondria by affinity purification paves the way to achieve long‐needed extra resolution of plant mitochondrial specialization. Here, guard cell and mesophyll cell mitochondria are tagged using Mito‐AP (Niehaus et al. 2020) in an attempt to compare their respective proteomes and unravel the peculiarities of guard cell mitochondria. Our data show that the pull‐down tag is cell type‐specific and that it is suited for the isolation of mitochondria from said cell types.

### 4.2 Protein Abundance Differences Between GDM and MDM May Be Underestimated due to Organellar Contaminations

LC–MS/MS analysis allows for pinpointing non‐mitochondrial contaminations in GDM and MDM fractions with high accuracy, and proteins from cytosol and plastids, but also from other compartments, are detected in Mito‐AP enriched organelle fractions. As such, it seems plausible that mitochondrial isolates from the targeted cell type may also be contaminated by mitochondria originating from off‐target cell types. Considering the low ratio of guard cells over mesophyll cells within a leaf, this may particularly affect the GDM fraction and may thus dilute abundance differences between GDM and MDM proteins. Although such contaminations obviously do not prevent identification of differently abundant proteins, in vivo ratios between GDM and MDM protein groups are expected to be higher than suggested by the data provided here.

### 4.3 Mitochondrial Energy Conversion in Guard Cells

Like any other cell type, guard cells require energy to perform their functions. Driven by ATP dephosphorylation, stomatal opening is achieved by an increased osmolality of guard cells due to an active transport of solutes and ions against a concentration gradient. Mitochondria are thus heavily invested in this energy‐demanding process. Based on reports of high numbers of mitochondria in guard cells (Allaway and Setterfield 1972; Willmer and Fricker 1996), as well as our results on the mitochondrial proteome and the enzymatic capacity of NADH oxidation in GDM as described here, we devise the following hypothesis on mitochondrial ATP production in guard cells:

1. Guard cell mitochondria utilize a different substrate mix for NADH production. A reduced rate of PR‐derived glycine oxidation in guard cells is (at least partly) compensated by a higher TCA‐cycle activity and the catabolism of Ala, Glu, and BCAAs.
2. Guard cell mitochondria use reduction equivalents for ATP synthesis more efficiently than their mesophyll counterparts. Statistical analysis shows a significantly lower abundance of alternative NAD(P)H dehydrogenases, but a higher abundance of proton‐pumping complex I and of the mobile electron carrier cytochrome *c* in GDM, indicating a more efficient use of NADH for ATP synthesis than in MDM.
3. Guard cell mitochondria respire at lower rates than mesophyll cell mitochondria. The ATP‐production capacity of mitochondria in guard cells is lower due to a reduced NADH‐oxidation capacity. At the same time, the ATP export capacity of GDM is decreased, together indicating a disposition toward a lower average ATP‐production rate per organelle. This reduced ATP‐production capacity is somewhat compensated for by a higher number of mitochondria within guard cells.

### 4.4 Why Do Guard Cell Mitochondria Require Lower Amounts of Protein Import Components?

**FIGURE 9:**
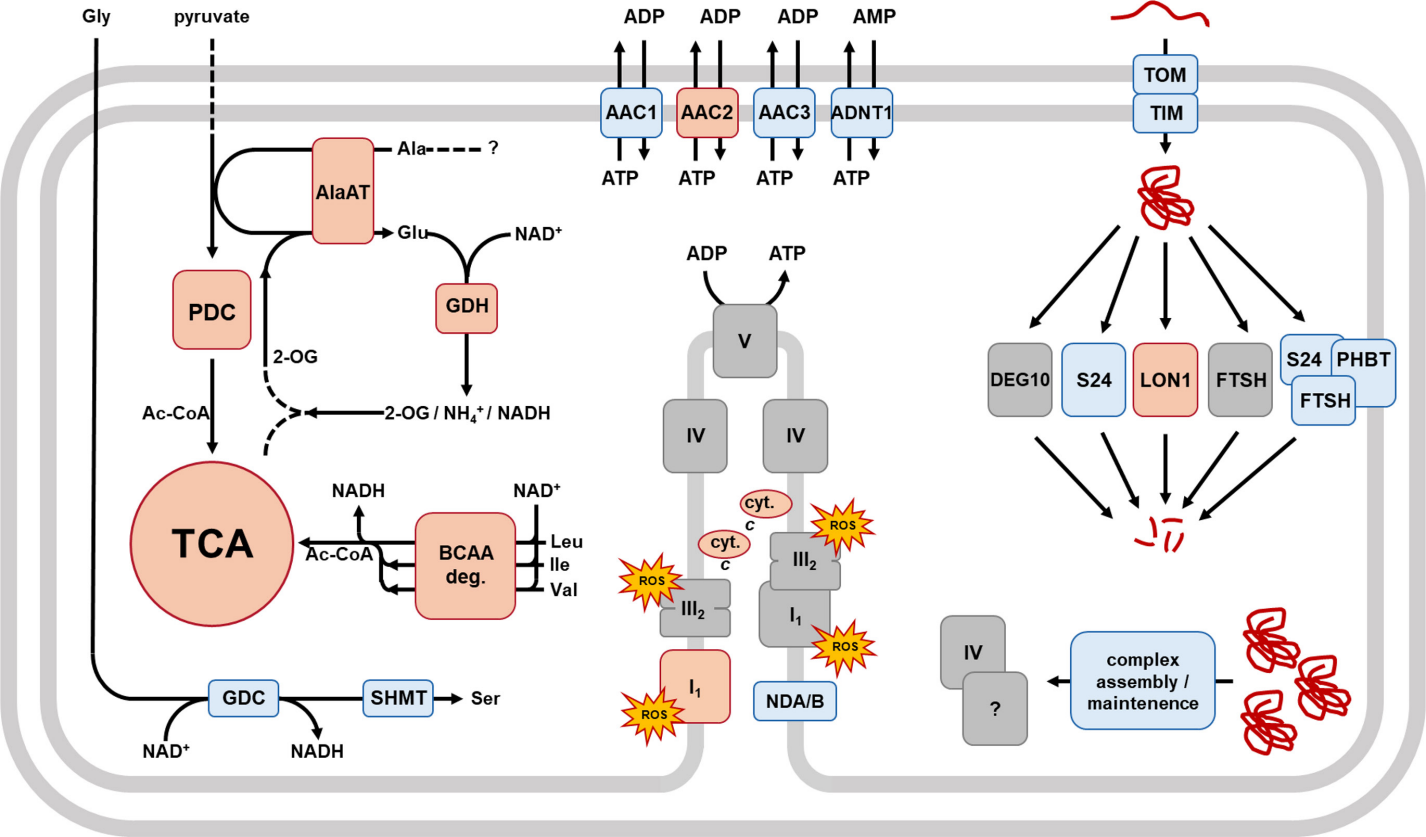
Graphical summary of physiological differences between guard cell and mesophyll mitochondria. Abundance differences of metabolic pathways are deduced from shotgun LC–MS/MS. Protein: Protein interactions of CI and CIII_2_, as well as of PHBT:FTSH:S24 peptidase, are based on BN‐PAGE and are shown by overlapping boxes. Significantly higher abundance in GDM when compared to MDM is indicated by red boxes, lower abundance in GDM is displayed in blue boxes. Non‐significant abundance differences between GDM and MDM are displayed in grey. Imported, assembled and degraded proteins are indicated by red wavy lines/wads. 2‐OG, 2‐oxoglutarate; AAC1‐3, mitochondrial ADP/ATP carrier; Ac‐CoA, acetyl coenzyme A; ADNT1, adenine nucleotide transporter 1; ADP, adenosine diphosphate; Ala, L‐alanine; AlaAT, alanine amino transferase 1; AMP, adenosine monophosphate; ATP, adenosine triphosphate; BCAA deg., branched chain amino acid catabolism; cyt *c*, cytochrome *c*‐1 and ‐2; DEG10, serine‐type endopeptidase; FTSH, matrix AAA^+^ type protease subunits FtsH3 and 10; GDC, glycine decarboxylase complex; GDH, NADH glutamate dehydrogenase subunit 1 and 2; Glu, L‐glutamate; Gly, L‐glycine; I_1_, monomeric respiratory complex I; III_2_, dimeric respiratory complex III; Ile, L‐isoleucine; IV, respiratory complex IV; Leu, L‐leucine; LON1, serine‐type Ion protease 1; NAD^+^, oxidized nicotinamide adenine dinucleotide; NADH, reduced nicotinamide adenine dinucleotide; NDA/B, internal and external alternative NAD(P)H dehydrogenases; PDC, mitochondrial pyruvate dehydrogenase complex; PHBT, prohibitin complex subunits; S24, peptidase S24; Ser, L‐serine; SHMT, serine hydroxymethyltransferase 1; TCA, tricarboxylic acid cycle; TIM, translocon of the inner mitochondrial membrane; TOM, translocon of the outer mitochondrial membrane; V, ATP‐synthase complex; Val, L‐valine.

Only a small proportion of mitochondrial proteins are synthesized *in organello*. Most mitochondrial proteins are produced in the cytosol and are post‐translationally imported into the organelles. Protein import, therefore, is a mainstay of mitochondrial biogenesis and proteome maintenance. This raises the question as to why the components of this pathway are of reduced abundance in GDM. In the absence of direct data on protein import and protein biosynthesis rates, we speculate that protein turnover in guard cell mitochondria happens at a slower pace than in mesophyll cell mitochondria (Figure 9). This theory is further supported by the studies from Hater et al. and Boussardon et al. (both this issue), who also report a reduction of protein import components and a lower abundance of protein groups associated with the translation machinery in guard cell mitochondria. In contrast, components involved in the degradation of mitochondrial proteins behave non‐uniformly. Together, this suggests a tight regulation of protein import and synthesis on one side, and protein turnover on the other side in organelles of both cell types.

### 4.5 Is the Enzymatic Composition of Guard Cell Mitochondria Geared for Reducing Respiratory ROS Production?

Respiratory complexes I, II, and III are major cellular producers of reactive oxygen species (ROS) (Huang et al. 2016), and mitochondrial ROS production increases under osmotic stress. It is currently unclear to what extent guard cell mitochondria experience osmotic stress upon influx of osmolytes into guard cells during stomatal opening and how this is affecting ROS production within these organelles. The reduced enzymatic activity of CI and the increased abundance of protein groups such as ISE1, ABC1K10A, and MST1 in GDM may serve to suppress mitochondrial ROS production under such conditions. Given that ROS also serve as signaling molecules in drought‐induced, ABA‐mediated stomatal closure by the indirect activation of anion channels in the plasma membrane (Postiglione and Muday 2020; Medeiros et al. 2020), mitochondrial ROS production during times of increased respiratory activity (which is needed to support stomatal opening) may thus interfere with stomatal control. We therefore speculate that the high numbers of mitochondria in guard cells showing a lower respiratory capacity on one side, and reduced amounts of NDB1, NDB2, and NDA1 within these organelles on the other side, may serve in adjusting the balance between ROS avoidance and NADH‐efficient ATP production within guard cells. A connection between mitochondrial ROS and reactive nitrogen species (RNS) production and altered stomatal response was indeed observed previously (Cvetkovska et al. 2014).

### 4.6 Future Research Directions

It is the nature of proteomic data that they provide only indirect insight into metabolic and cellular processes. They are thus not suited to mirror the actual in vivo situation with high accuracy. However, proteomic analyses often provide excellent starting points for new research directions, since they offer untargeted and unbiased data covering a multitude of metabolic pathways and cellular processes. Enabled by the recent advancements in organellar isolation, the proteomic data provided by this study provide new insights but require additional work to test the hypotheses developed here. This includes a detailed functional analysis on the ostensibly higher preference of guard cell mitochondria for the oxidation of some amino acids, as well as detailed studies on electron transfer along the respiratory chain in these organelles. Potentially adverse effects on mitochondrial functions by osmolyte influx into the organelles are also a topic that may deserve further attention. Finally, little is currently known about the regulatory process governing guard cell mitochondrial metabolism. Apart from these topics, the proteomic dataset provided here will most likely entail special features of guard cell mitochondria that are not touched upon in this manuscript. We invite readers to examine our data and to develop new ideas on how these organelles fulfil their functions and contribute towards balancing leaf transpiration and carbon fixation.

## 5 Conclusions

Substrate utilization for NADH production in guard cell mitochondria differs from that of their mesophyll cell counterparts, relying to a higher degree on non‐PR amino acids (such as Ala, Glu, and BCAAs) as well as the TCA cycle. The flow of electrons within these specialised mitochondria is expected to concentrate more on the route along the classical electron transfer complexes and less along the route provided by plant‐specific alternative respiratory enzymes. Guard cell mitochondrial energy conversion (in terms of ATP/O ratio) is thus expected to be more efficient than that of mesophyll cell mitochondria. However, ATP production rates of individual guard cell mitochondria may be lower, potentially reducing the risk of ROS production, which in turn may reduce indirect interference of mitochondrial ATP production with stomatal opening.

## Author Contributions

M.H. and M.N. designed the Mito‐AP constructs. M.N. performed cloning, plant transformation, and the generation of stable expressing Mito‐AP lines. N.D. and N.M.E. performed confocal fluorescence microscopy. N.D. performed Mito‐AP, shotgun proteomics, BN‐PAGE, NADH‐oxidation assays, phenotypic analysis, and data analysis. H.E. coordinated and supervised the project and analyzed data. All authors were involved in writing the manuscript.

## Conflicts of Interest

The authors declare no conflicts of interest.

## Supporting information


**Figure S1:** Identification of Mito‐AP tagged organelles in H500 seedlings.
**Figure S2:** Phenotypic comparison of Mito‐AP lines.
**Figure S3:** Influence of Mito‐AP construct expression on cellular proteomes of H500, H972, and H973.
**Figure S4:** Contribution of identified protein groups to subcellular compartments in crude cellular leaf extracts and mitochondrial isolates of Col‐0 plants and Mito‐AP lines.
**Figure S5:** Distribution of identified mitochondrial protein groups within Mito‐AP fractions.
**Figure S6:** Protein abundance differences between protein groups involved in metabolite transport across the inner mitochondrial membrane in guard cell and mesophyll cell mitochondria.
**Figure S7:** Protein abundance differences of OXPHOS components in guard cell and mesophyll cell mitochondria.
**Figure S8:** Protein abundance differences between protein groups involved in protein modification as well as OXPHOS assembly and maintenance in guard cell and mesophyll cell mitochondria.
**Figure S9:** Electrophoretic mobility of prohibition isoforms, FTSH proteins, and peptidase S24 in BN gels.

**Data S1:** Supporting Information.

**Table S1:** All mitochondrial protein groups identified in this study.

**Table S2:** Commonly and uniquely detected protein groups in GDM, MDM, and TLM.

**Table S3:** Protein abundance differences between guard cell derived mitochondria (GDM) and mesophyll derived mitochondria (MDM).

**Table S4:** Abundance of identified mitochondrial protein groups in fractions of the NADH‐oxidation assay after affinity purification of total leaf mitochondria (H500), mesophyll derived mitochondria (H973) and guard cell derived mitochondria (H972).

## Data Availability

All LC–MS/MS raw files acquired in this study, as well as the corresponding MaxQuant search engine results can be downloaded under ftp://massive‐ftp.ucsd.edu/v09/MSV000097408/. Clustered abundance profiles of all mitochondrial proteins identified in the TLM, MDM, and GDM BN gels can be inspected at our complexomemap portal under the following address: https://complexomemap.de/projects‐guard/.
