# Supplementary material for: Proteomic Analysis Infers Optimized ATP‐Production in Guard Cell Mitochondria
Source: Physiol Plant. 2025 Sep 26;177(5):e70529. doi: 10.1111/ppl.70529 (PMC12464808; doi:10.1111/ppl.70529)
Supplement: Supplementary file 1 — Figure S1: Identification of Mito‐AP tagged organelles in H500 seedlings. Figure S2: Phenotypic comparison of Mito‐AP lines. Figure S3: Influence of Mito‐AP construct expression on cellular proteomes of H500, H972, and H973. Figure S4: Contribution of identified protein groups to subcellular compartments in crude cellular leaf extracts and mitochondrial isolates of Col‐0 plants and Mito‐AP lines. Figure S5: Distribution of identified mitochondrial protein groups within Mito‐AP fractions. Figure S6: Protein abundance differences between protein groups involved in metabolite transport across the inner mitochondrial membrane in guard cell and mesophyll cell mitochondria. Figure S7: Protein abundance differences of OXPHOS components in guard cell and mesophyll cell mitochondria. Figure S8: Protein abundance differences between protein groups involved in protein modification as well as OXPHOS assembly and maintenance in guard cell and mesophyll cell mitochondria. Figure S9: Electrophoretic mobility of prohibition isoforms, FTSH proteins, and peptidase S24 in BN gels. [file PPL-177-e70529-s001.pdf]

Proteomic analysis infers optimized ATP-production in guard cell mitochondria

Noah Ditz, Markus Niehaus, Nieves Medina Escobar, Marco Herde, Holger Eubel

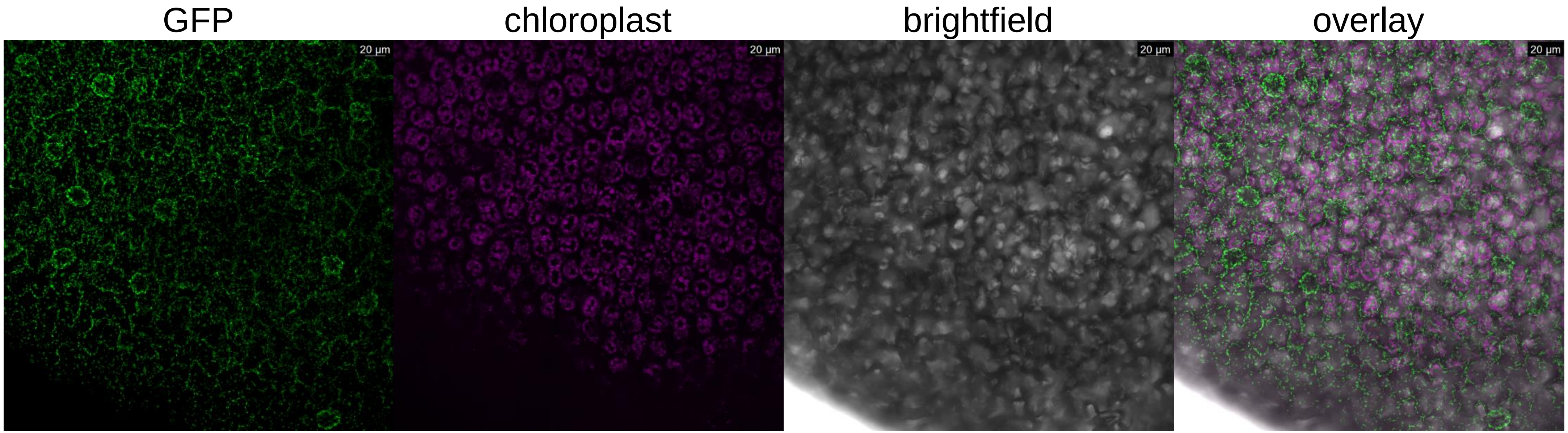

Supp. Figure 1. Identification of Mito-AP tagged organelles in H500 seedlings. Confocal fluorescence microscopy of *A. thaliana* plants carrying the H500 Mito-AP construct. Seedlings were placed on a petri dish containing filter paper soaked in 2 mL 0.5x MS-media. After 5 days under long day conditions, seedlings were transferred onto glass slides and cotyledons were analyzed by confocal fluorescence microscopy. Images were taken from three biological replicates, of which representatives examples are displayed. Panels from left to right, GFP signal originating from the Mito-AP construct, chlorophyll autofluorescence, brightfield, overlay of all three channels. Scale bars are 20 µm.

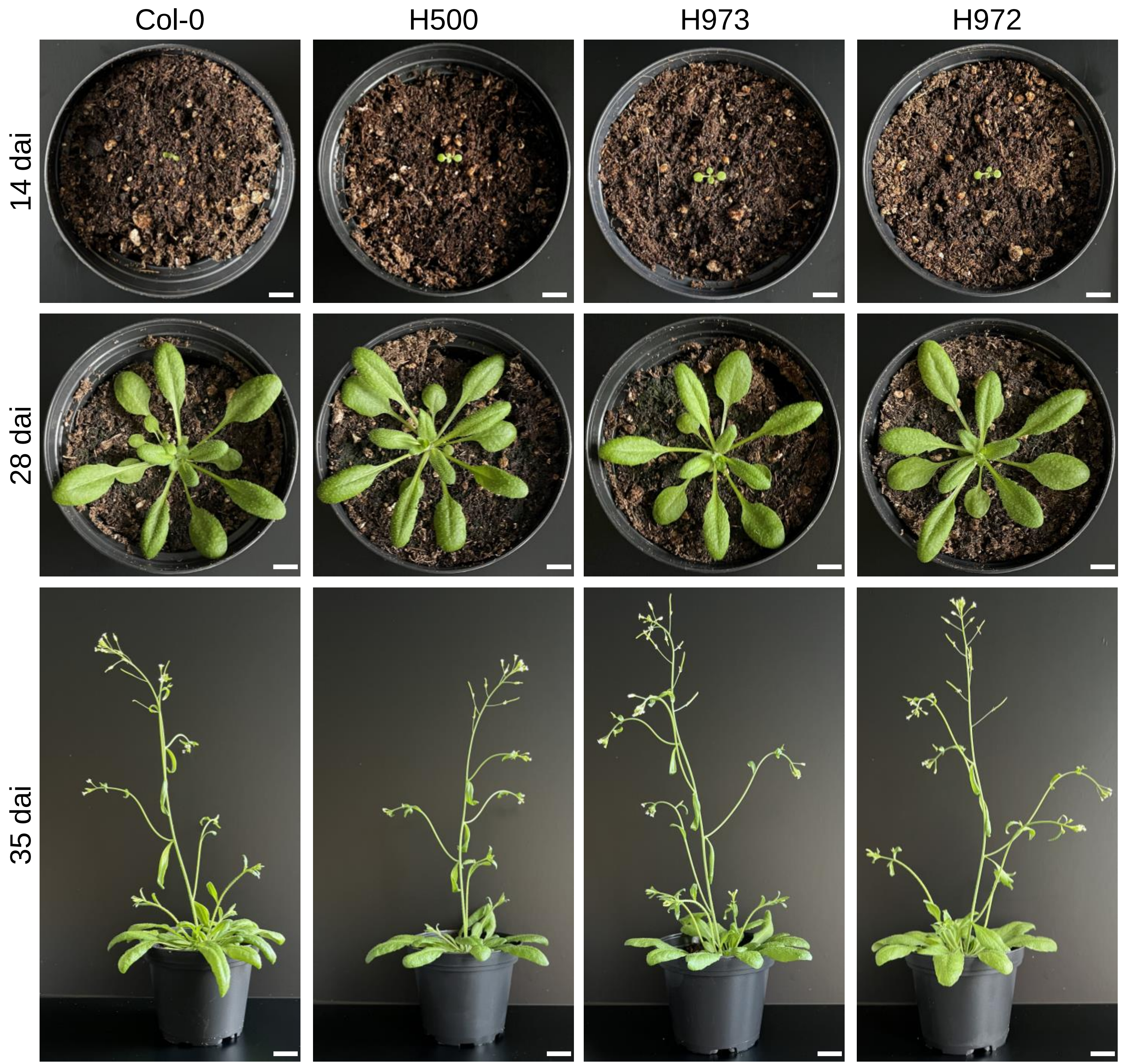

Supp. Figure 2. Phenotypic comparison of Mito-AP lines *A. thaliana* plants were stably transformed with the Mito-AP construct under control of different, cell type specific promotors. Seeds of homozygous T_2 plants of H500, H973, and H972 as well as Col-0 WT plants were imbibed and stratified for 3 days at 4° C, before being placed in a long day (16 h light / 8 h darkness) conditions under a PPFD of 120 µE. Temperature was 22° C during the day and 20° C in the night, relative humidity was kept at 65%. Pictures were taken 14, 28 and 35 days after imbibition (dai). Five independent biological replicates were screened and representative images for each line were chosen. Scale bars in the upper two panels represent 1 cm, bottom scale bars represent 2 cm.

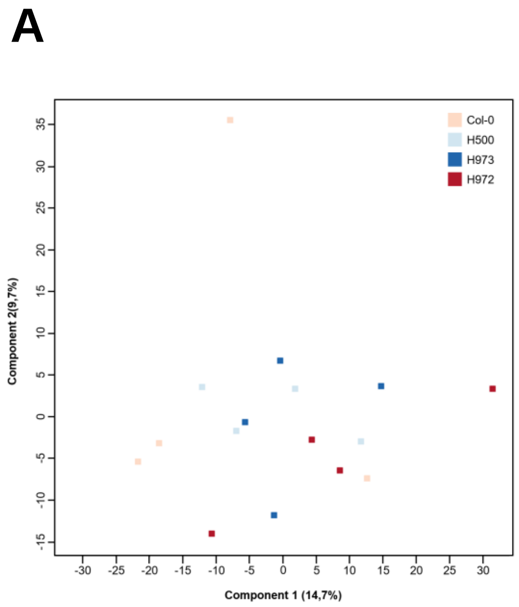

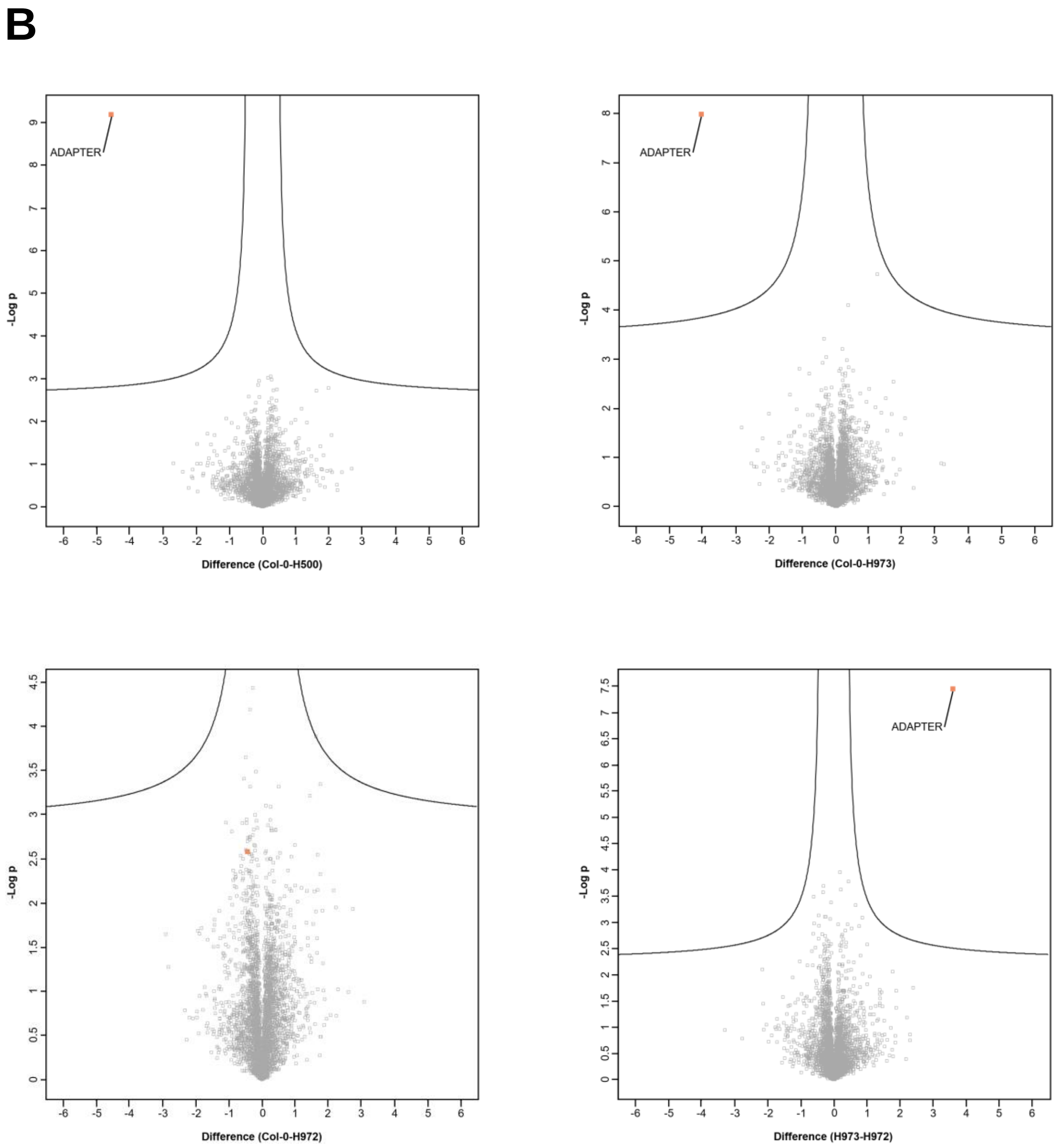

Supp. Figure 3. Influence of Mito-AP construct expression on cellular proteomes of H500, H972, and H973. Crude leaf protein extracts of four biological replicates of Col-0, H500, H973 and H972 plants were analyzed by LC-MS/MS. A, principal component analysis (PCA). Col-0 samples are shown in pink, H500 in light blue, H973 in dark blue and H972 in red. B, Abundance differences of protein groups between Col-0 and Mito-AP cell extracts (top left, top right, and bottom left panel), as well as between H972 and H973 Mito-AP lines (bottom right panel). X-axis, difference between log_2-transformed LFQ-values; y-axis, -log p-values. Proteins located above the black lines fulfill the selection criteria for a significantly different abundance (FDR ≤ 0.05; S0 ≥ 0.1). Data points representing the ADAPTER protein are colored orange.

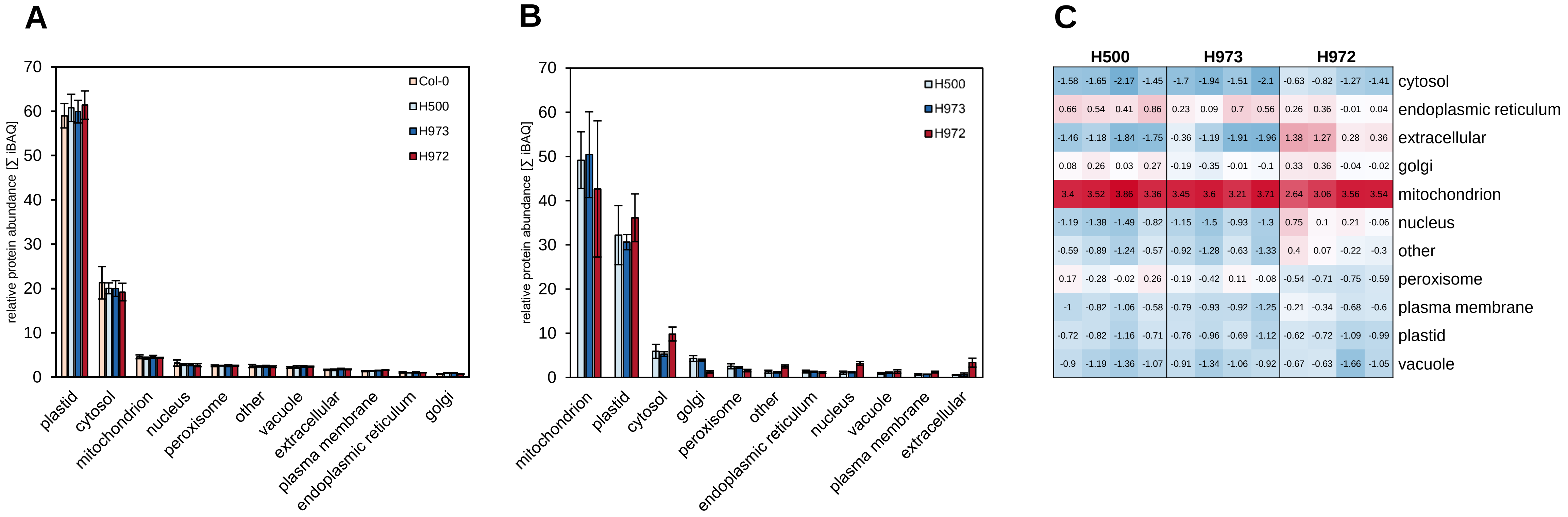

Supp. Figure 4. Contribution of identified protein groups to subcellular compartments in crude cellular leaf extracts and mitochondrial isolates of Col-0 plants and Mito-AP lines. A**,** Rosette leaves of four week old *A. thaliana* plants grown under long day conditions were homogenized to obtain crude leaf protein extracts from four biological replicates. LC-MS/MS analysis was performed and subcellular localization of identified protein groups was assigned using the SUBAcon algorithm. Intensity based abundance quantification values (iBAQs) were cumulated for all proteins assigned to the same compartment to assess the relative contribution of each compartment to the total leaf proteome. Proteins that were assigned to several compartments by the SUBAcon algorithm are listed here as 'other'. B, protein abundance was calculated in the same way for samples after Mito-AP to estimate mitochondrial enrichment. C, Log_2 transformed protein abundance differences of subcellular compartments between crude extract and Mito-AP fractions. Protein abundance of different subcellular compartments relative to the total protein abundance was calculated for individual biological replicates before and after Mito-AP of the H500, H973 and H972 lines. Higher protein abundance after Mito-AP is indicated in red, lower protein abundance is indicated in blue. Log_2 fold changes of protein abundance are given in the individual cells. Mito-AP lines are given at the top, subcellular compartments to the right.

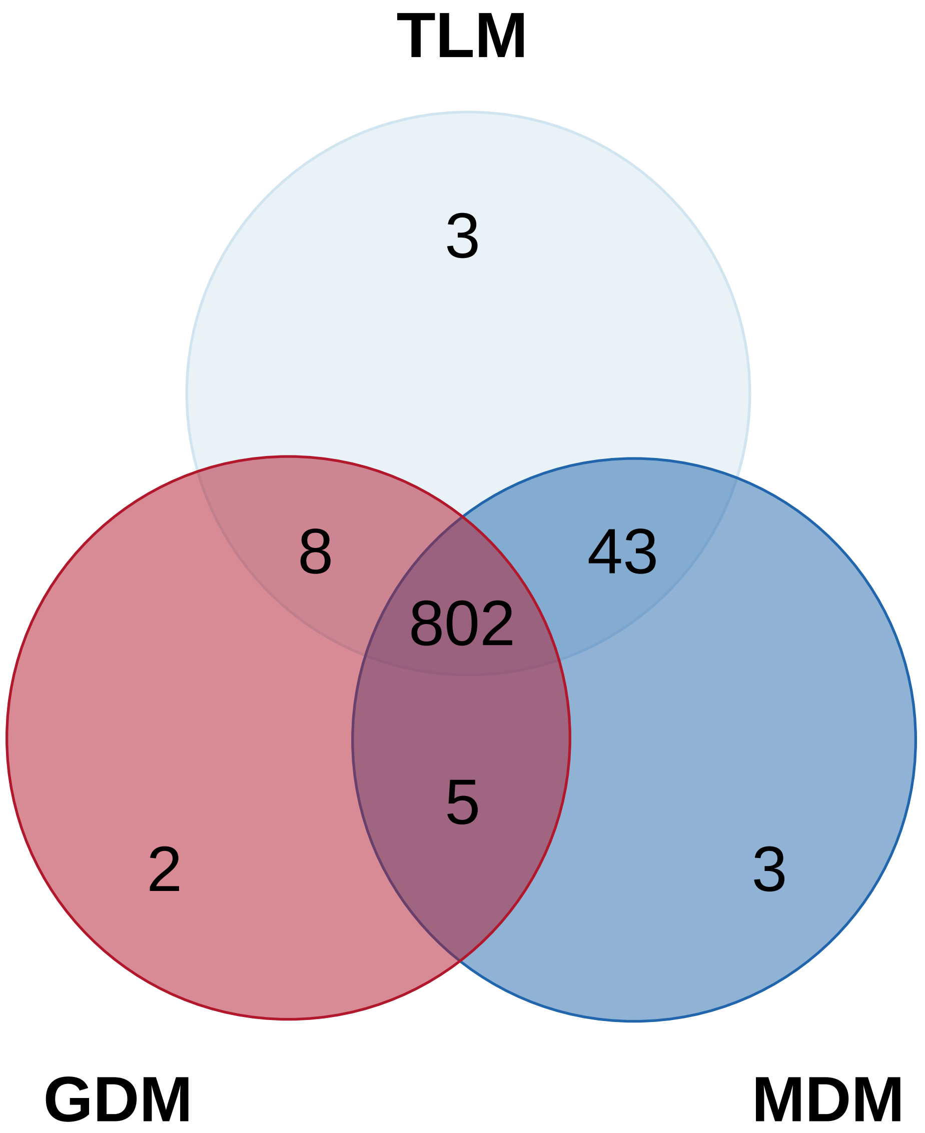

Supp. Figure 5. Distribution of identified mitochondrial protein groups within Mito-AP fractions. Four replicates of total leaf mitochondria (TLM), guard cell derived mitochondria (GDM), and mesophyll derived mitochondria (MDM) (as obtained by Mito-AP) were analyzed by shotgun proteomics. Considering protein groups identified in at least a single replicate, identification of mitochondrial entries (according to the SUBAcon algorithm; Hooper et al. 2014) is compared.

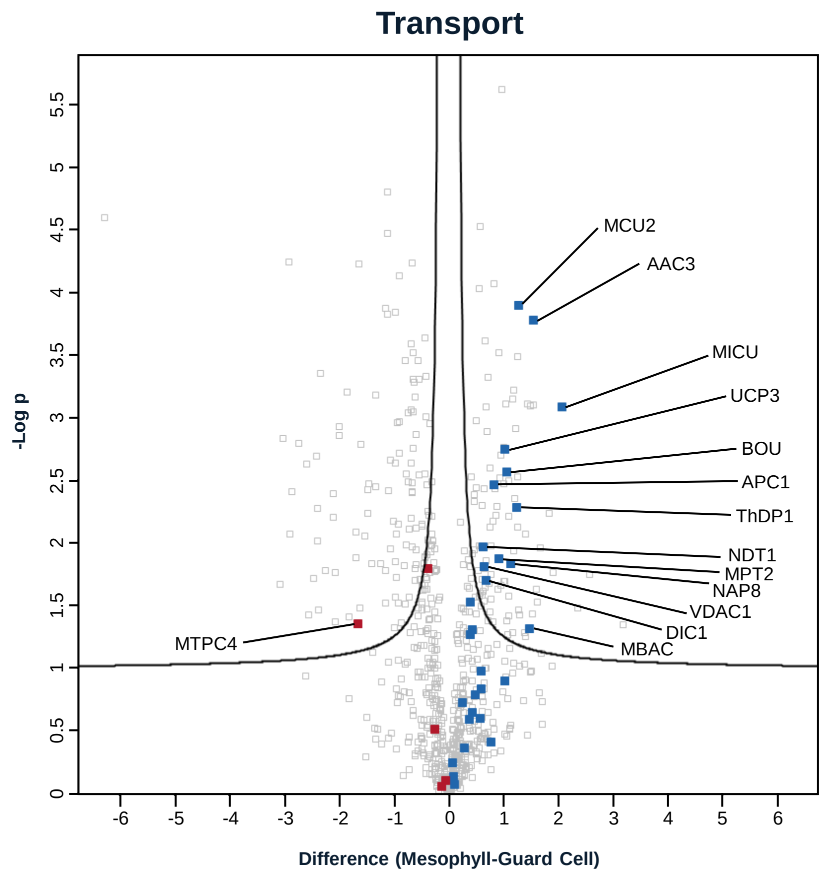

Supp. Figure 6. Protein abundance differences between protein groups involved in metabolite transport across the inner mitochondrial membrane in guard cell and mesophyll cell mitochondria. After normalization, log_2-transformed LFQ values of protein groups assigned to mitochondria by the SUBAcon algorithm were used to assess differences in the mitochondrial proteomes of guard cells and mesophyll cells by statistical testing. X-axis, difference between log_2-transformed LFQ-values; y-axis, -log p-values. Proteins located above the black lines fulfill the selection criteria for a significantly different abundance (FDR ≤ 0.05; S0 ≥ 0.1). Protein groups displayed in dark red are of higher abundance in guard cell derived mitochondria (GDM), protein groups shown in blue are of higher abundance in mesophyll derived mitochondria (MDM).

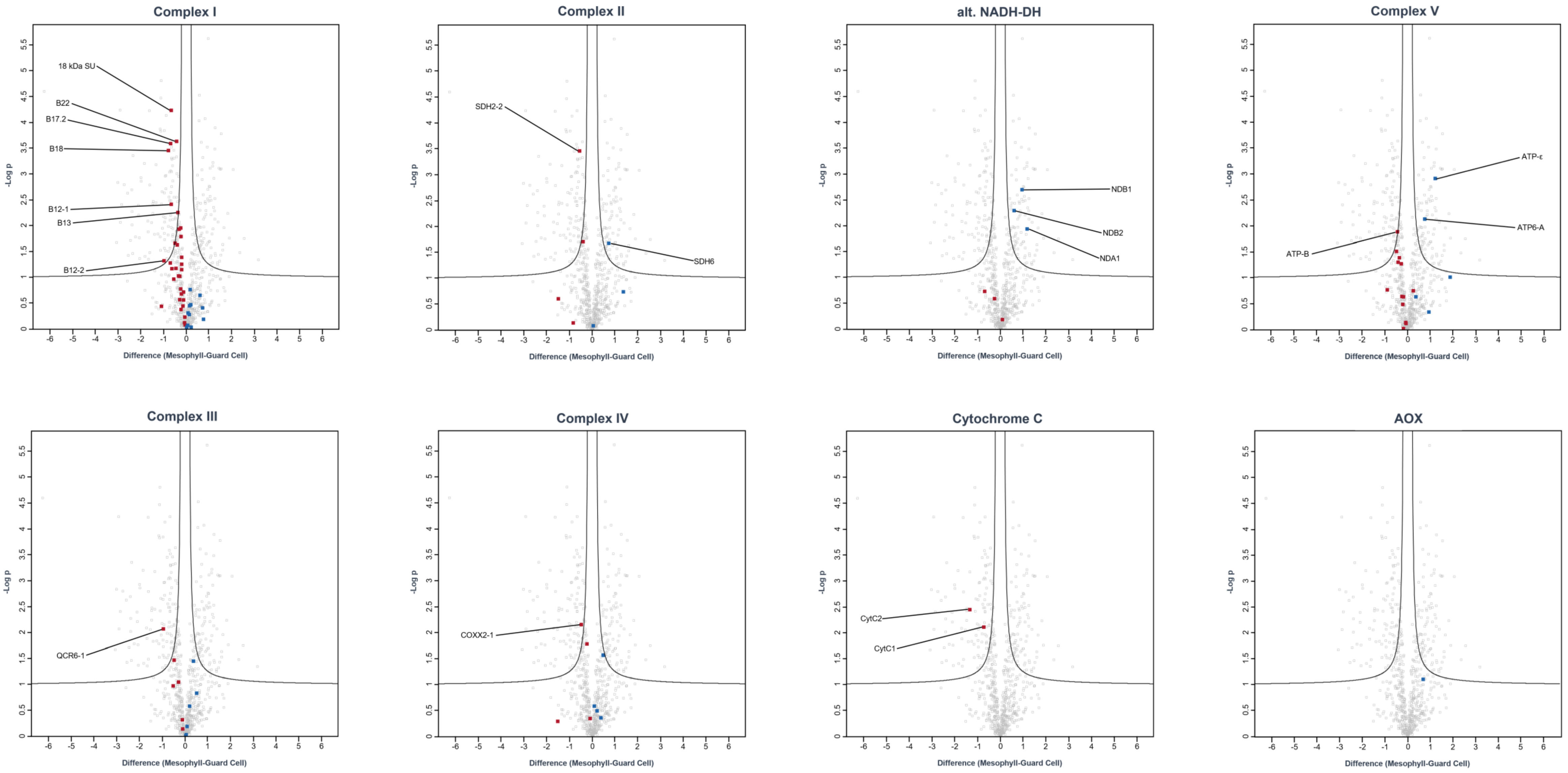

Supp. Figure 7. Protein abundance differences of OXPHOS components in guard cell and mesophyll cell mitochondria. After normalization, log2-transformed LFQ values of protein groups assigned to mitochondria by the SUBAcon algorithm were used to assess differences in the mitochondrial proteomes of guard cells and mesophyll cells by statistical testing. x-axis, difference between log2-transformed LFQ-values; y-axis, -log p-values. Proteins located above the black lines fulfill the selection criteria for a significantly different abundance (FDR, 0.05; S0, 0.1). Protein groups displayed in dark red are of higher abundance in GDM, protein groups shown in blue are of higher abundance in MDM.

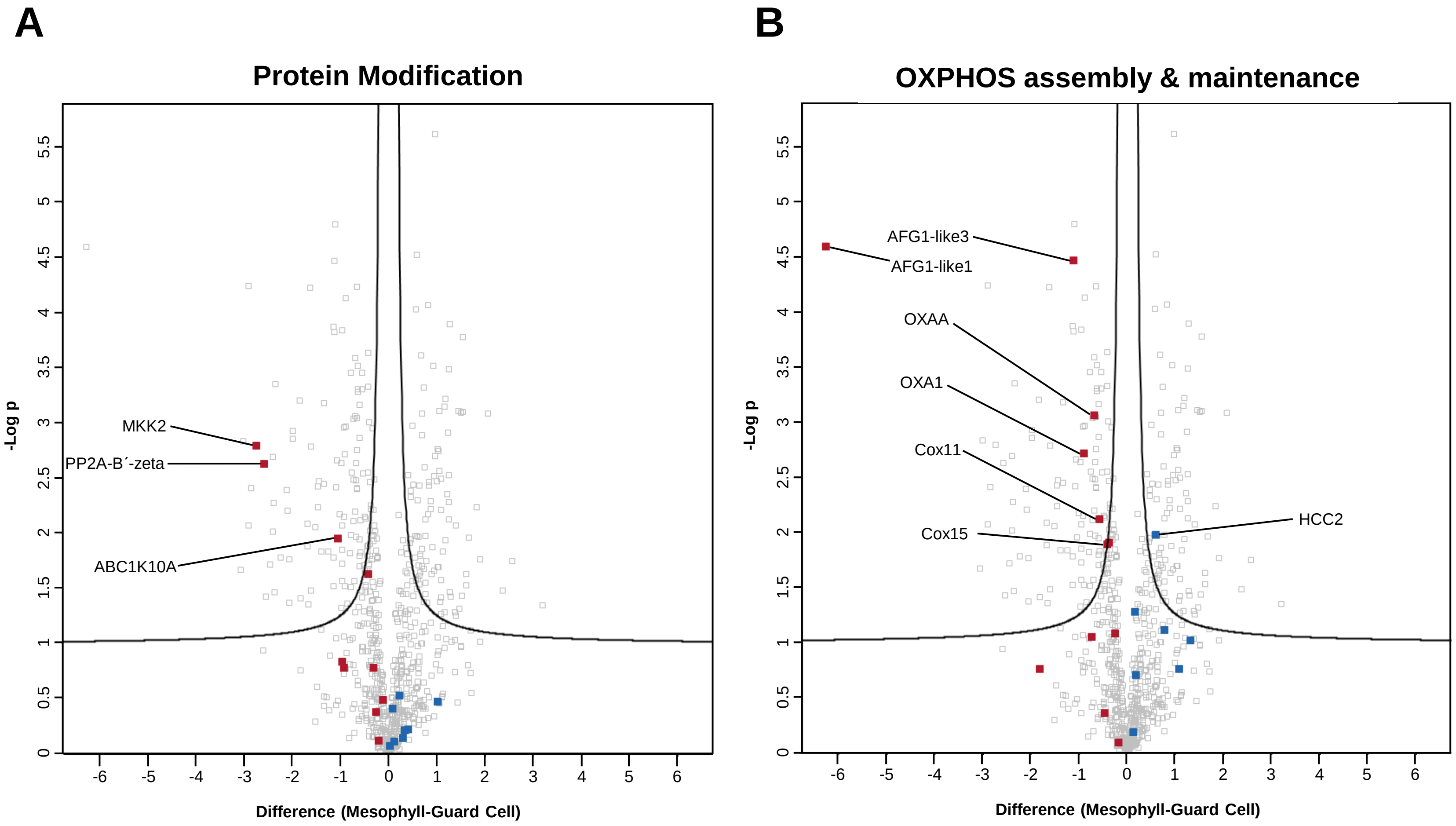

Supp. Figure 8. Protein abundance differences between protein groups involved in protein modification as well as OXPHOS assembly and maintenance in guard cell and mesophyll cell mitochondria. After normalization, log2-transformed LFQ values of protein groups assigned to mitochondria by the SUBAcon algorithm were used to assess differences in the mitochondrial proteomes of guard cells and mesophyll cells by statistical testing. x-axis, difference between log2-transformed LFQ-values; y-axis, -log p-values. Proteins located above the black lines fulfill the selection criteria for a significantly different abundance (FDR, 0.05; S0, 0.1). Protein groups displayed in dark red are of higher abundance in GDM, protein groups shown in blue are of higher abundance in MDM.

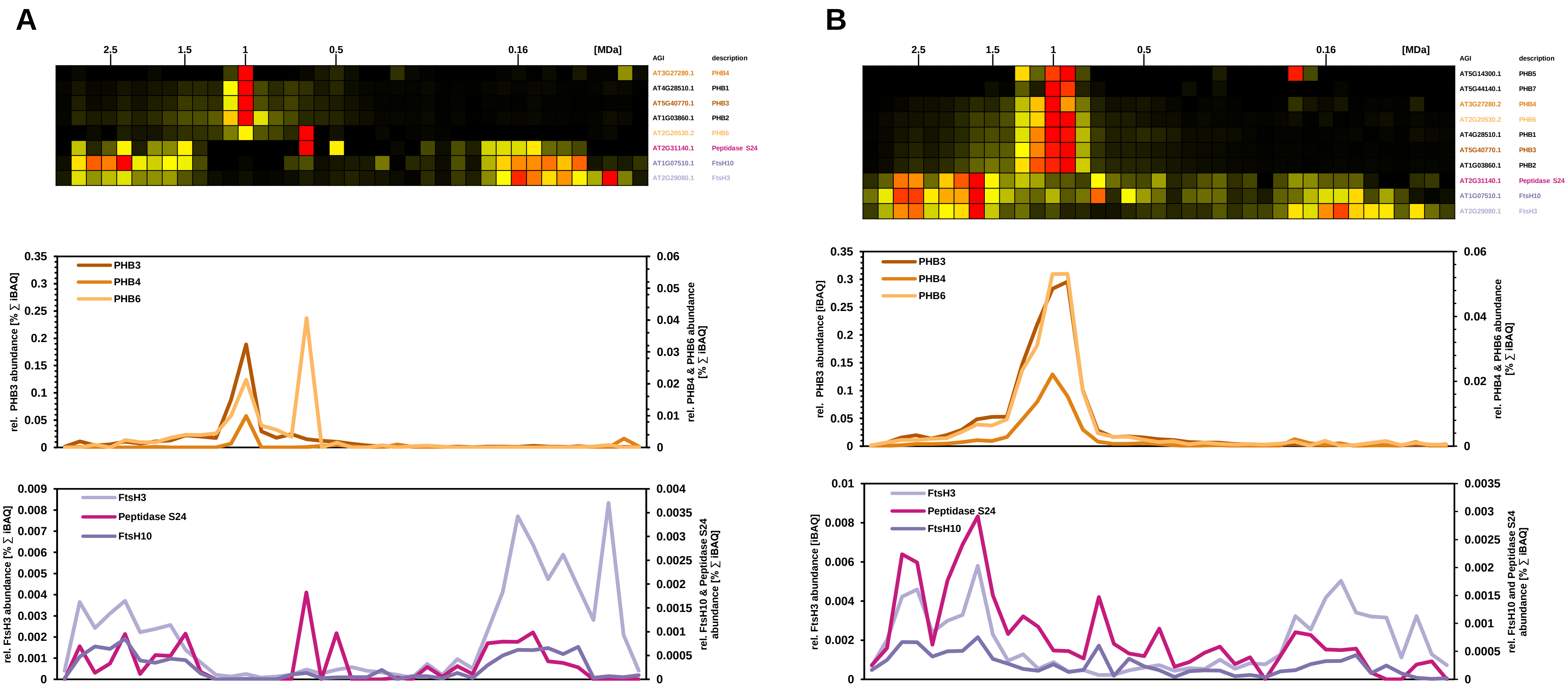

Supp. Figure 9. Electrophoretic mobility of prohibitin isoforms with FTSH proteins and peptidase S24 in BN gels. Protein abundance profiles of prohibitin (PHB) isoforms, FTSH subunits and peptidase S24 in GDM (A) and MDM (B) along a BN gel lane are shown in a color gradient. Maximum normalized protein abundance [iBAQ] is depicted in red, medium and low relative protein abundance in orange and yellow, respectively. Lack of detection is indicated as black squares. Arabidopsis gene identifier (AGI) and protein description are given to the right of each heatmap. Molecular masses are indicated on top of heatmaps. Prohibitin isoforms, FTSH subunits, and S24 peptidase were selected manually and re-clustered. Complete heatmaps showing clustered abundance profiles of all mitochondrial proteins are available at https://complexomemap.de/projects-guard/. Middle panels show abundance distribution along the BN gel for PHBs involved in formation of the mitochondrial FTSH protease complex in GDM (left) and MDM (right), whereas lower panels show abundance distributions of FtsH proteins and peptidase S24. Protein abundance in the graphs is displayed relative to the cumulated mitochondrial iBAQ values of GDM and MDM, to better illustrate stoichiometries between the proteins shown in top heatmaps.
