# Supplementary material for: Proteomic Analysis Infers Optimized ATP‐Production in Guard Cell Mitochondria
Source: Physiol Plant. 2025 Sep 26;177(5):e70529. doi: 10.1111/ppl.70529 (PMC12464808; doi:10.1111/ppl.70529)
Supplement: Supplementary file 2 — Data S1: Supporting Information. [file PPL-177-e70529-s005.pdf]

Suppl. Methods

Shotgun Proteomics

For all shotgun proteomic analyses, four independent biological replicates were used. Samples were treated with 250 µl SDT buffer (4% [w/v] SDS, 0.1 M DTT, 0.1 M Tris-HCl, pH 7.6), incubated for 1 h at 60 °C and 1000 rpm and stored at -20 °C. The collected replicates where then together subjected to the single pot, solid phase enhanced sample preparation (SP3) protocol (Hughes et al. 2019) with minor adjustments for samples originating from plant material (Mikulášek et al. 2021). Samples were centrifuged at 20000xg for 10 min and the supernatant was incubated for 10 min in an ultrasonication bath. Samples were again centrifuged for 10 min at 20000xg and 30 µl of the supernatants were transferred to new tubes. Alkylation of cysteine residues was performed by adding iodoacetamide to a final concentration of 20 mM. After incubation for 30 min in the dark, fresh DTT was added to a final concentration of 5 mM. Each sample was mixed with 30 µl bead stock consisting of hydrophilic and hydrophobic magnetic beads (Sera-Mag carboxylate-modified beads, 10 µg $µl^{-1}$ hydrophilic solids no. 241521050250, 10 µg $µl^{-1}$ hydrophobic solids no. 441521050250, GE Healthcare Life Sciences, Düsseldorf, Germany). Protein binding to the beads was induced by addition of ethanol yielding a final concentration of 50% [v/v], followed by incubation for 10 min at 24 °C and 1000rpm in an orbital shaker. Beads were pelleted on a magnet for 2 min, the supernatant was removed and proteins were washed in 80% [v/v] ethanol. After four wash steps, the mixture was transferred to low protein binding (LPB) reaction tubes and beads were pelleted for 2 min in a magnetic field. The supernatant was discarded and proteins were digested by adding 1 µg of sequencing-grade modified trypsin (V5111, Promega) in 50 mM ammonium bicarbonate and overnight incubation at 37 °C and 1000rpm in an orbital shaker. Beads were pelleted for 2 min in a magnetic field and supernatants were transferred to new tubes. Beads were rinsed with 60 µl 50 mM ammonium bicarbonate and pelleted again for 2 min before supernatants were pooled with those of the previous step. Proteolysis was stopped by addition of 1% [v/v] formic acid to the pooled supernatants. Peptides were desalted on 50 mg Sep-Pak tC18 columns (WAT054960, Waters; Eschborn, Germany) and quantified afterwards using the Pierce Quantitative Colorimetric Peptide Assay Kit (Thermo Fisher Scientific, Dreieich, Germany) following the manufacturer's instructions. Final peptide concentrations were adjusted to 200 ng $µl^{-1}$ in 0.1% [v/v] formic acid (FA).

Samples were stored at 4 °C in the autosampler of a nanoElute2 UHPLC coupled to a timsTOF Pro MS (both Bruker, Bremen, Germany) before analysis. Two hundred nanograms of peptides were directly loaded on a reversed-phase C18 analytical column (Aurora Ultimate 25 cm x 75 µm, 1.7 µm particle size, 120 Å pore size; IonOpticks, Fitzroy, Australia) equipped with a zero-dead-volume (ZDV) emitter. Separation of peptides was achieved at 50 °C using a 60 min acetonitrile (ACN) gradient ranging from 2% [v/v] to 37% [v/v] ACN in the presence of 0.1% [v/v] formic acid (FA). Afterwards, the ACN

concentration was increased to 95% [v/v] over 2 min and kept at this value for 6 min to clear the column of non-peptide compounds.

Ionization of peptides took place at a temperature of 180 °C and a capillary voltage of 1600 V. The scan range was defined from 100-1700 m/z using the PASEF scan mode with ion mobilities (expressed as 1/K0) starting at 0.85 V s^{-1} cm^{-2} and ending at 1.3 V s^{-1} cm^{-2}. The ramp time was set to 100 ms at a ramp rate of 9.42 Hz, resulting in four PASEF ramps with a total cycle time of 0.53 s. Maximum charge was set to five and the target intensity was set to 14500 using an intensity threshold of 1200. Collision energies were set to 27 eV for a 1/K0 of 0.85 V s^{-1} cm^{-2} and to 45 eV for a 1/K0 of 1.3 V s^{-1} cm^{-2} and were linearly interpolated between these values. Active exclusion was set to 0.4 min. Acquired MS/MS-spectra were queried against an in-house modified Tair10 database containing protein sequences considering organellar RNA-editing using the MaxQuant (Cox and Mann, 2008) software version 2.4.10.0, enabling the 'no fractions' option. Default settings of the software were used as summarized in the following: Oxidation of methionine residues and acetylation of N-termini were selected as variable modifications, carbamidomethylation of cysteines as a fixed modification. Trypsin (P) was selected as protease with a maximum of two missed cleavages. Maximum charge was set to five, maximum peptide mass was defined as 4600 Da with a minimum peptide length of seven and a maximum peptide length of 25 for unspecific searches. The 'match between runs' function was enabled. PSM and protein FDR were set to 0.01. Absolute and relative protein abundance was computed by enabling the 'iBAQ' and 'LFQ' functions.

Non-normalized intensity based absolute quantitation (iBAQ) values (Schwanhäusser et al. 2011) were used for assessment of organelle enrichment during Mito-AP, since these values allow for 'vertical' operations, including the generation of cumulated abundance data for proteins belonging to the same cellular compartment. Label free quantitation (LFQ) values (Cox and Mann 2008) were instead used for horizontal comparisons of protein abundance (i.e. for assessing the abundance of each individual protein across all samples).

Principal Component Analysis (PCA) and Volcano Plots were produced from MaxQuant LFQ-values using the Perseus software version 2.1.2.0 (Tyanova et al., 2016). To compare mitochondrial proteomes, protein groups that were assigned to other subcellular compartments than mitochondria by the SUBAcon algorithm (Hooper et al. 2014) were manually removed before analysis. Proteins that were identified only by one modified peptide were removed, as were peptides for which only versions carrying a variable modification. LFQ values were then log_2-transformed and proteins were functionally assigned according to an in-house curated MapMan annotation (Usadel et al., 2009). Protein groups were classified as 'quantifiable' if they were identified at least two times within each of the three sample groups. Missing values were imputed using the 'Replace missing values from normal distribution' function with a width of 0.3 and a down shift of 1.8. It is important to note that LFQ value

calculation already involves a normalization step. However, given that the degree of mitochondrial enrichment varied between the individual isolates, a re-normalization step was performed using the 'width adjustment' function of Perseus. Volcano plots (requiring a FDR of ≤ 5%, S0 ≥ 0.1) as well the PCA-analysis are based on this re-normalised set of mitochondrial protein abundance.
